# Widespread exon skipping triggers degradation by nuclear RNA surveillance in fission yeast

**DOI:** 10.1101/gr.185371.114

**Published:** 2015-06

**Authors:** Danny A. Bitton, Sophie R. Atkinson, Charalampos Rallis, Graeme C. Smith, David A. Ellis, Yuan Y.C. Chen, Michal Malecki, Sandra Codlin, Jean-François Lemay, Cristina Cotobal, François Bachand, Samuel Marguerat, Juan Mata, Jürg Bähler

**Affiliations:** 1 University College London, Research Department of Genetics, Evolution and Environment and UCL Cancer Institute, London WC1E 6BT, United Kingdom;; 2 Université de Sherbrooke, Department of Biochemistry, Sherbrooke, Quebec J1H 5N4, Canada;; 3 Department of Biochemistry, University of Cambridge, Cambridge CB2 1QW, United Kingdom

## Abstract

Exon skipping is considered a principal mechanism by which eukaryotic cells expand their transcriptome and proteome repertoires, creating different splice variants with distinct cellular functions. Here we analyze RNA-seq data from 116 transcriptomes in fission yeast (*Schizosaccharomyces pombe*), covering multiple physiological conditions as well as transcriptional and RNA processing mutants. We applied brute-force algorithms to detect all possible exon-skipping events, which were widespread but rare compared to normal splicing events. Exon-skipping events increased in cells deficient for the nuclear exosome or the 5′-3′ exonuclease Dhp1, and also at late stages of meiotic differentiation when nuclear-exosome transcripts decreased. The pervasive exon-skipping transcripts were stochastic, did not increase in specific physiological conditions, and were mostly present at less than one copy per cell, even in the absence of nuclear RNA surveillance and during late meiosis. These exon-skipping transcripts are therefore unlikely to be functional and may reflect splicing errors that are actively removed by nuclear RNA surveillance. The average splicing rate by exon skipping was ∼0.24% in wild type and ∼1.75% in nuclear exonuclease mutants. We also detected approximately 250 circular RNAs derived from single or multiple exons. These circular RNAs were rare and stochastic, although a few became stabilized during quiescence and in splicing mutants. Using an exhaustive search algorithm, we also uncovered thousands of previously unknown splice sites, indicating pervasive splicing; yet most of these splicing variants were cryptic and increased in nuclear degradation mutants. This study highlights widespread but low frequency alternative or aberrant splicing events that are targeted by nuclear RNA surveillance.

Splicing is a fundamental step in gene expression, which determines the information content of messenger RNAs (mRNAs). It is a highly accurate process that relies on the spliceosome to identify sequence signals and remove noncoding introns from precursor mRNAs (pre-mRNAs) and thus generate translatable mRNAs. In its simplest form, splicing requires several intronic sequence elements for intron excision, including splice donor and acceptor sites, a branch-site, and a polypyrimidine tract (Wang and Burge 2008). Another layer of complexity, alternative splicing, results in the production of multiple transcripts from a single gene, some of which may be condition- or tissue-specific (Wang et al. 2008). These transcripts, referred to as alternative splice variants, carry nonconsecutive combinations of exons and are thought to be a major source of transcriptome and proteome diversity in multicellular eukaryotes (Demir and Dickson 2005; Keren et al. 2010). Alternative splicing can also alter post-translational modifications (Merkin et al. 2012) or affect translation and protein localization (Stamm et al. 2005). Splicing is tightly integrated with gene regulation (Braunschweig et al. 2013; Bentley 2014) and can control gene expression via nonsense-mediated (Le Hir et al. 2003; Guan et al. 2006) or spliceosome-mediated (Volanakis et al. 2013) decay pathways (NMD and SMD, respectively).

Alternative splice variants may arise via single or multiple exon-skipping or intron-retention events or via alternative 5′- or 3′-splice sites (Wang et al. 2008). The extent of alternative splicing in the human transcriptome is potentially immense, with >90% of genes showing more than one isoform (Wang et al. 2008). Alternative splicing can be governed by sequence elements such as intronic and exonic splicing enhancers or silencers, which are recognized by RNA-binding proteins that facilitate or inhibit splicing (Fairbrother et al. 2002; Yeo et al. 2007). A “splicing code,” which dictates the final composition of a given transcript in a given condition, remains elusive. It is now apparent that splice site selection is not only dictated by sequence information but also by intricate interplays between chromatin, RNA polymerase II, and the spliceosome (Alexander and Beggs 2010; Schwartz and Ast 2010).

Unspliced and partially spliced transcripts can be deleterious (Egecioglu et al. 2012; Sayani and Chanfreau 2012), and aberrant splicing is implicated in human diseases (Cooper et al. 2009). Cells therefore use several nuclear and cytoplasmic quality-control pathways to degrade faulty transcripts. In human and yeast, the nuclear exosome acts as the first and main line of protection (Garneau et al. 2007; Schmid and Jensen 2008; Houseley and Tollervey 2009). The exosome is a multiprotein complex containing nine noncatalytic core subunits, which interact in the nucleus with the two catalytic subunits, Rrp6 and Dis3, that confer 3′-5′ exoribonuclease and endoribonuclease activities, respectively (Houseley et al. 2006; Garneau et al. 2007; Schmid and Jensen 2008; Schneider and Tollervey 2013). RNA degradation by the exosome is facilitated by polyadenylation activity of the TRAMP complex (LaCava et al. 2005; Schmidt and Butler 2013), by helicase activity (Houseley et al. 2006; Schmidt and Butler 2013), and by decapping and deadenylation (Garneau et al. 2007; Arribas-Layton et al. 2013). In budding yeast, unspliced and partially spliced transcripts, as well as transcripts with abnormal exon skipping, are actively degraded by the nuclear exosome, the 5′-3′ nuclear exonuclease Rat1 (Dhp1 in fission yeast), or the RNase III endonuclease Rnt1 (Egecioglu et al. 2012; Sayani and Chanfreau 2012).

Defective transcripts that evade degradation by nuclear surveillance may still be degraded by cytoplasmic surveillance pathways (Sayani and Chanfreau 2012). In yeast, cytoplasmic degradation occurs via the core exosome associated with Dis3 (Houseley et al. 2006; Schmid and Jensen 2008), via the 5′-3′ cytoplasmic exonuclease Xrn1 (Exo2 in fission yeast) (Wood et al. 2002; Sayani and Chanfreau 2012), or via the 3′-5′ Dis3L2 RNA decay pathway (Malecki et al. 2013). Cytoplasmic mRNA degradation by the exosome is initiated by deadenylation and assisted by the helicase activity of the SKI complex (Anderson and Parker 1998; Schmid and Jensen 2008). Cytoplasmic degradation can also be triggered by the NMD pathway that recognizes premature stop codons (Sayani et al. 2008; Kawashima et al. 2014) or by the nonstop decay (NSD) pathway that identifies missing stop codons (Frischmeyer et al. 2002). Partially spliced transcripts are degraded by the Dbr1-dependent pathway, which recognizes intronic lariat intermediates (Hilleren and Parker 2003).

Alternative splicing is widely thought to greatly augment protein diversity. A recent analysis of human transcriptomes, however, has revealed that most protein-coding genes express only one dominant transcript that contributes to the proteome in multiple tissues (Gonzàlez-Porta et al. 2013). In contrast to tissue-specific gene expression programs, alternative splicing is much less conserved and often lineage-specific (Barbosa-Morais et al. 2012; Merkin et al. 2012). Furthermore, stochastic splicing errors can increase transcript isoform diversity (Melamud and Moult 2009; Pickrell et al. 2010). These findings raise the possibility that a considerable fraction of alternative splice variants simply reflect splicing errors without any cellular function or with functions other than alternative protein production.

To explore this hypothesis, we analyzed transcriptomes under diverse environmental, physiological, and genetic perturbations in the fission yeast *Schizosaccharomyces pombe*. Approximately 47% of the *S. pombe* genes contain annotated introns, and the splicing machinery is conserved (Käufer and Potashkin 2000). Limited evidence suggests alternative splicing via intron retention (Moldón et al. 2008; Duncan and Mata 2014) or exon skipping (Awan et al. 2013) for a few *S. pombe* genes, but some exon-skipping events have been attributed to splicing errors (Bitton et al. 2014). We show here that widespread but rare exon-skipping events, and many other unannotated splicing events, mostly result in transcripts that are actively degraded via the nuclear exosome and Dhp1. These observations indicate that alternative splicing by exon skipping in fission yeast represents aberrantly spliced transcripts that are largely destined for degradation in the nucleus.

## Results

We analyzed transcriptomes of wild-type *S. pombe* strains under the following physiological and environmental conditions: vegetative growth in minimal and rich media, stationary phase, quiescence, heat stress, and meiotic differentiation. We also analyzed transcriptomes of several mutants with defects in the following RNA processing and transcriptional processes/complexes: core and catalytic subunits of the RNA exosome, nuclear and cytoplasmic 5′-3′ exonuclease, TRAMP and SKI complexes, NMD and NSD, mRNA decapping, poly(A) binding, cytoplasmic deadenylation, RNA splicing and debranching, RNAi, nucleosome remodeling, and RNA polymerase II. Including biological repeats, our analysis encompassed RNA-seq data from 116 transcriptomes from different physiological and genetic perturbations, including both published and original data. A detailed overview of this data set is provided in Supplemental Table S1.

### Exon-skipping events are widespread but rare

**Figure 1.**
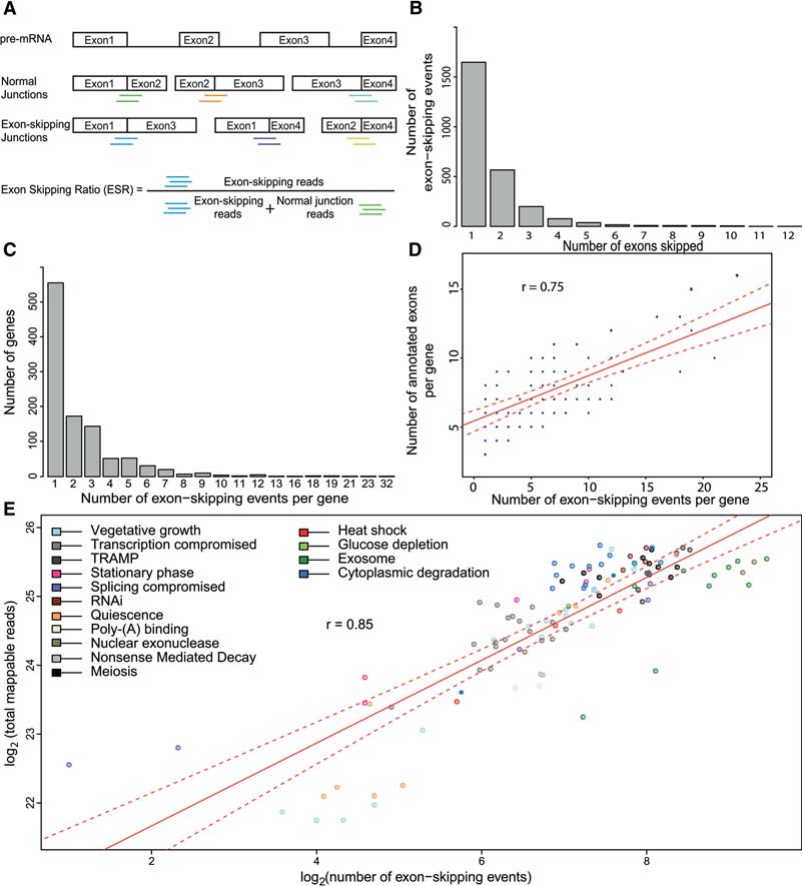
Identification and characterization of exon-skipping events using RNA-seq. (*A*) Scheme showing pre-mRNA (*top*) along with all possible normally spliced mRNAs and exon-skipping isoforms. The colored lines *below* the junctions represent diagnostic reads for normal exon–exon junctions (5′-3′; green, orange, cyan), and for exon-skipping junctions (5′-3′; blue, purple, light green). These diagnostic reads were used to calculate the exon-skipping ratio (ESR) and to identify splice isoforms. (*B*) Total number of exon-skipping events (*y*-axis) as a function of number of exons being skipped (*x*-axis; ranging from one to 12 skipped exons during a single event); 2574 skipping events are shown. (*C*) Maximum number of exon-skipping events recorded per gene (*x*-axis) as a function of gene number (*y*-axis). (*D*) Correlation between numbers of exon-skipping events per gene and numbers of annotated exons in the same gene. In total, 1063 genes were binned based on number of detected exon-skipping events and number of their annotated exons. The size of data points was scaled according to number of genes in each bin, i.e., normalized by number of genes in the genome containing a corresponding number of exons; (r) Pearson's correlation coefficient; (red line) fitted regression; (dotted red lines) 0.95 confidence levels. (*E*) Correlation between sequencing depth and number of exon-skipping events. Strains were grouped and color coded according to cellular function or condition tested (Supplemental Table S1); (r) Pearson's correlation coefficient; (red line) fitted regression; (dotted red lines) 0.95 confidence levels.

To identify sequence reads that represent exon-skipping events among the multiple samples analyzed, we generated a database containing all theoretical exon–exon junctions for all genes in *S. pombe*. We aligned all sequence reads against this junction database and simultaneously against the reference genome, retaining only reads mapping to a single location (3,676,121,463 mappable reads in total) (Fig. 1A). For the exon-skipping analysis, we used all exon–exon junction reads for both normal (consecutive) and exon-skipping splicing (Fig. 1A).

About 2.2% of the mappable reads originated from exon–exon junctions (82,232,577 reads) (Supplemental Fig. S1), but only 0.001% of the mappable reads were diagnostic for exon-skipping events (44,616 reads). We initially considered all “skipping reads” that represent single or multiple exon-skipping events in a given transcript and condition. This analysis identified 2574 distinct exon-skipping events in 1063 genes (Supplemental Table S2), including 21 previously documented events (Supplemental Table S3; Awan et al. 2013). As the fission yeast genome contains only 1375 genes with at least three annotated exons, our finding implies that ∼77.3% of these genes are alternatively spliced by exon skipping.

The majority of these exon-skipping events only involved the skipping of one exon, and the number of exon skipping decreased with increasing numbers of skipped exons (Fig. 1B). Moreover, genes typically only contained one exon-skipping event, and the number of genes greatly decreased with increasing numbers of exon-skipping events per gene (Fig. 1C). The number of exon-skipping events per gene was correlated with the number of exons (Fig. 1D); hence, the likelihood of exon skipping increased with higher numbers of introns to be spliced. The number of exon-skipping reads showed marginal positive correlations with the lengths of the 5′- and 3′-flanking introns (Supplemental Fig. S2A,B) and weak inverse correlations with the lengths of the skipped exons and the entire spliced regions (Supplemental Fig. S2C,D). Accordingly, Egecioglu et al. (2012) reported that small exons are more likely to be skipped in budding yeast. Furthermore, exon skipping only marginally correlated with the expression levels of the corresponding transcripts (Supplemental Figs. S3, S4). On the other hand, the number of exon-skipping events strongly increased with sequencing depth (Fig. 1E). This finding reflects that transcripts carrying exon-skipping information are rare, and their identification therefore heavily depends on sequencing depth. Taken together, we conclude that exon-skipping events are infrequent yet widespread in the fission yeast transcriptome. The number of exon-skipping events per gene increases with increasing exon numbers, but is only weakly affected by expression level and length of the spliced region. These findings are consistent with exon-skipping events largely representing splicing errors.

### Exon skipping increases in nuclear RNA degradation mutants and during meiotic differentiation

**Figure 2.**
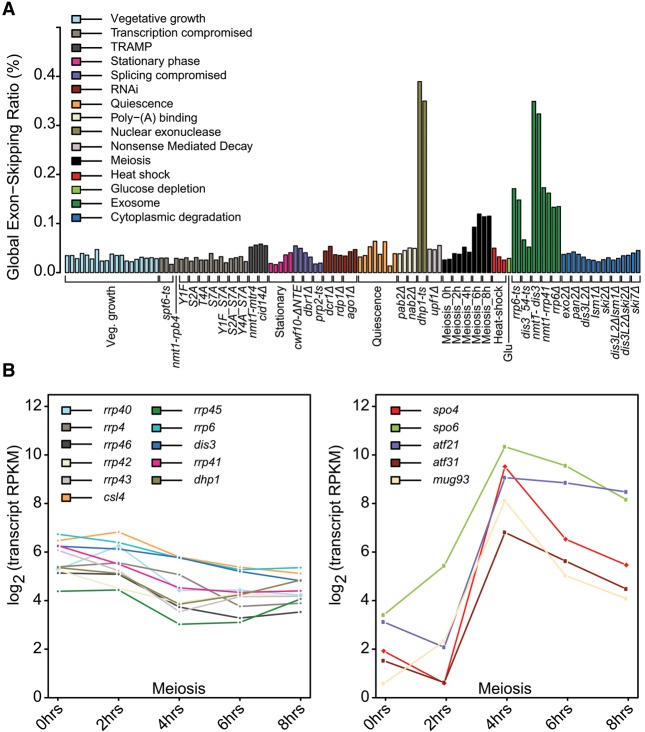
Exon-skipping events are rare but accumulate in nuclear surveillance mutants and meiosis. (*A*) Sample-specific global exon-skipping ratio (ESR), reflecting the proportion of exon-skipping reads among total exon–exon junction reads. Physiological conditions or mutants as indicated *below* were grouped and color coded according to cellular function or condition tested (Supplemental Table S1). (*B*) RNA-seq expression profiles of selected transcripts during meiotic differentiation. (*Left*) Exosome subunit and nuclear exonuclease transcripts. (*Right*) Selected transcripts known to increase during meiosis (Mata et al. 2002). Mean expression of two biological replicates is shown at each time point (RPKM, reads per kilobase per million). The decrease in nuclear-exosome transcripts is significant (*P*-adjust < 0.05, DESeq) (Anders and Huber 2010).

To further investigate the possibility that exon-skipping events represent splicing errors, we calculated the global exon-skipping ratio (ESR), i.e., the proportion of all skipping reads among all exon–exon junction reads for each sample (Fig. 1A). This analysis revealed a significant enrichment of exon skipping in the nuclear-exosome mutant, *rrp6*, and the nuclear-exonuclease mutant, *dhp1* (*P* < 2.2 × 10^−16^, Cochran-Mantel-Haenszel test, Bonferroni corrected) (Fig. 2A). Also, other exosome subunit mutants (*rrp41* and *dis3*), components of both nuclear and cytoplasmic exosome complexes, exhibited a pronounced increase in exon-skipping events. Strains with a mutated *dis3* RNase II domain (*dis3_54-ts*) showed a significant but more moderate increase in exon skipping compared to *dis3*, *rrp6*, or *rrp41* mutants, in line with the documented reduction in exonuclease activity in *dis3_54-ts* cells (Murakami et al. 2007). In the case of *dis3* and *rrp41* mutants, it is not possible to determine whether a faulty nuclear or cytoplasmic exosome gave rise to the accumulation of exon-skipping events. However, given the dramatic increase in exon skipping when the function of the nuclear proteins Dhp1 and Rrp6 were impaired and the lack of increased exon skipping in several cytoplasmic degradation mutants, it seems most likely that impaired nuclear-exosome activity led to the observed increase in *dis3* and *rrp41* mutants.

More modest, but significant, increases in exon skipping were also evident in some of the other mutants and conditions tested (Fig. 2A), e.g., mutants with impaired TRAMP complex (*nmt1-mtr4*, *cid14*Δ; *P* < 1.3 × 10^−13^) or with defective splicing (*cwf10*-Δ*NTE*; *P* < 2.0 × 10^−12^). Notably, mutants with impaired cytoplasmic degradation showed no significant increase in exon skipping (Fig. 2A), and the NMD pathway mutant showed only a subtle increase (*upf1*Δ; *P* < 1.1 × 10^−11^). These results indicate that the majority of exon-skipping transcripts are degraded by the nuclear exosome and Dhp1 exonuclease, although other pathways might play minor or backup roles.

Exon-skipping events also significantly increased during late meiotic differentiation, 6–8 h after induction of *pat1-114-*driven meiosis (*P* < 2.2 × 10^−16^) (Fig. 2A), corresponding to meiosis II and sporulation (Mata et al. 2002). Many of the core and catalytic nuclear-exosome transcripts decreased during meiosis, particularly at 4 h following meiotic induction, with a mean decrease of ∼2.7-fold (Fig. 2B). In contrast, transcripts known to be regulated during meiosis increased as expected (Fig. 2B). This finding raises the possibility that the increased exon skipping in late meiosis could result from lower nuclear surveillance activity.

Taken together, our analysis reveals that exon-skipping events increase most when nuclear RNA degradation pathways are compromised and during late stages of sexual differentiation when transcript levels for components of the surveillance machinery decrease. These results further suggest that most exon-skipping events represent aberrantly spliced transcripts that are actively degraded by nuclear RNA surveillance.

### Exon-skipping variants are stochastic across physiological conditions and unlikely to be translated

**Figure 3.**
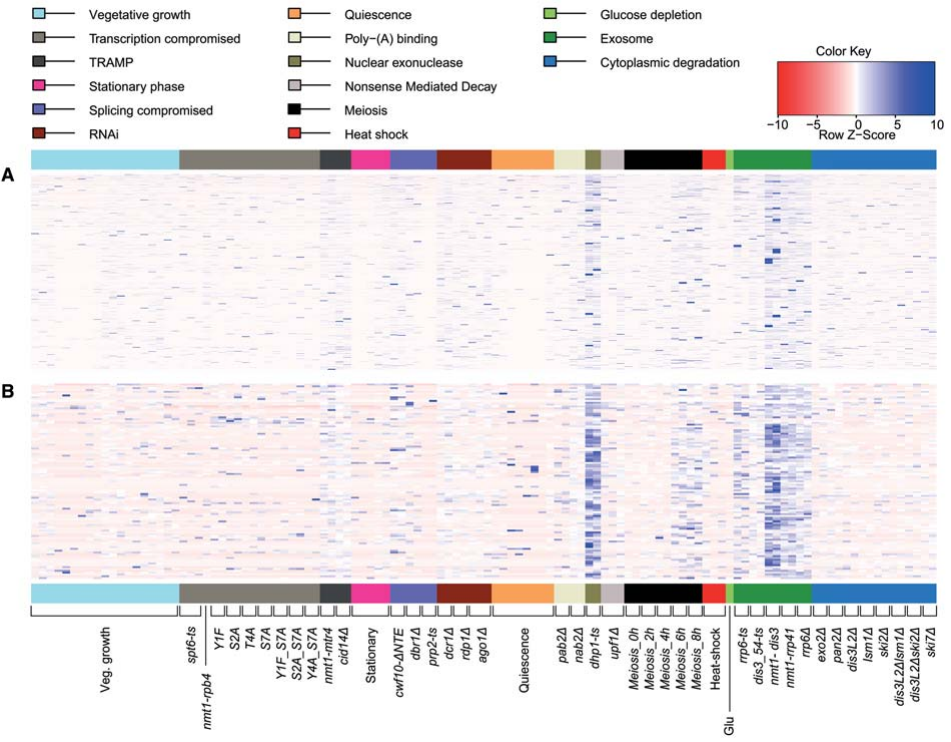
Splice variant-specific, local exon-skipping ratios (ESR) highlighting the stochastic nature of exon skipping. (*A*) Heatmap representing 2504 exon-skipping events for which both normal splicing and exon-skipping reads were identified in one or more samples (ratios provided in Supplemental Table S4). Physiological conditions or mutants as indicated *below* were grouped and color coded according to cellular function or condition tested. Maximum distance between rows (exon-skipping events) was determined using the “dist” function in R (method “maximum”), followed by hierarchical clustering using the “hclust” function. Row *Z*-score: Ratios in each row were scaled by subtracting the mean of the row from each value, followed by division of resultant values by the SD of the row, i.e., (local ESR value − row mean)/row SD. (*B*) As in *A* but showing local ESR for the high confidence set (111 exon-skipping events supported by nine or more exon-skipping reads in one or more samples). Only 107 exon-skipping events for which both normal and exon-skipping reads were identified in one or more samples are shown (Supplemental Table S5).

The global ESR revealed overall enrichment of exon-skipping events in specific samples (Fig. 2A). However, this global analysis would not uncover any alternatively spliced variants that might increase in relative abundance in specific samples, i.e., potential isoform switching. To find such transcripts, we computed the local, variant-specific ESR (Fig. 1A; Supplemental Table S4). The local ESR represents the proportion of exon-skipping events among all splicing events at a given locus. As for the global ESR, this analysis showed that exon skipping was more pronounced in nuclear degradation mutants and during late meiotic stages (Fig. 3A). However, the local ESR was typically close to 0 (average across entire data set: ∼0.004), and was often variable between biological replicates (Fig. 3A; Supplemental Table S4). These results are consistent with exon skipping reflecting random splicing errors.

RNA-seq experiments are known to suffer from Poisson noise (or counting/shot noise) when read counts are low (Anders and Huber 2010). Since we initially considered any skipping read as evidence for exon skipping, it is possible that the observed variability reflects Poisson noise. Therefore, to mitigate the effect of low-read counts, we applied a series of sample-specific skipping-read cutoffs when considering a putative exon-skipping event. As expected, the number of putative exon-skipping events decreased with increasing skipping-read counts (Supplemental Fig. S5A). Notably, ∼51% of the skipping reads in our data set (22,817/44,616) originated from only 111 exon-skipping events (∼4.3% of total exon-skipping events), occurring in 108 genes (Supplemental Table S5). These events were supported by more than eight skipping reads in at least one sample, referred to below as the high-confidence exon-skipping set. We could not detect any functional characteristic or DNA sequence features among these exons that might explain why they are much more frequently skipped. The few genes that are much more prone to exon skipping might be truly alternatively spliced. As the analyses below show, however, even this high-confidence set most likely represents splicing errors.

The local ESR appeared less variable when only considering the high-confidence exon-skipping set (Fig. 3B). Even among this set, however, the local ESR only showed reproducibly higher values in cells with compromised RNA degradation and during late meiotic differentiation (Fig. 3B). Moreover, no significant functional enrichment was evident, neither for the entire set of 1063 genes in which exon skipping occurred nor for the high-confidence set of 108 genes. We further ranked the high-confidence exon-skipping set based on local ESR and reproducibility across the 46 physiological conditions tested, excluding genetically perturbed samples (ESR ≥0.1, followed by ranking based on numbers of samples that met the threshold). Only eight exon-skipping events displayed an ESR ≥0.1 in at least nine of the 46 samples (Supplemental Table S5). The local ESR of these highest-abundance events was still further increased in nuclear RNA surveillance mutants, however, suggesting that even these high-confidence exon-skipping events are subject to degradation.

We then explored the likelihood that exon-skipping transcripts are translated. Across the entire data set, only 904 of 2574 skipped exons were divisible by three with respect to nucleotide number (i.e., did not change the reading frame); this subset did not exhibit any higher number of skipping reads (∼35%, *P* > 0.05) (Supplemental Fig. S5B). Also, the high-confidence exon-skipping set was not enriched for skipped exons divisible by 3 (34%), and the eight most frequent exon-skipping events also displayed a pattern close to random expectation (3/8; 37.5%). Thus, most exon-skipping transcripts are unlikely to produce a functional protein if the same start codon is being used (Magen and Ast 2005). We also analyzed ribosome-protected mRNA fragments (RPFs) from ribosome profiling data (Duncan and Mata 2014). These data only contained 123 RPFs across 72 exon-skipping junctions out of 325,103 junction RPFs in total (∼0.04%) (Supplemental Fig. S6). The numbers of RPFs across exon-skipping junctions were typically much lower than the numbers of RPFs across the corresponding normal splice junctions (Supplemental Table S6). Such low read counts do not allow distinguishing between translated regions and contaminants in the RPF samples (Duncan and Mata 2014). Overall, the ribosome profiling data do not support that exon-skipping transcripts are translated. Taken together, we conclude that exon skipping in fission yeast is largely random, infrequent, and unlikely to be functional or translated in any of the physiological conditions analyzed.

### RNA surveillance masks splicing errors

**Figure 4.**
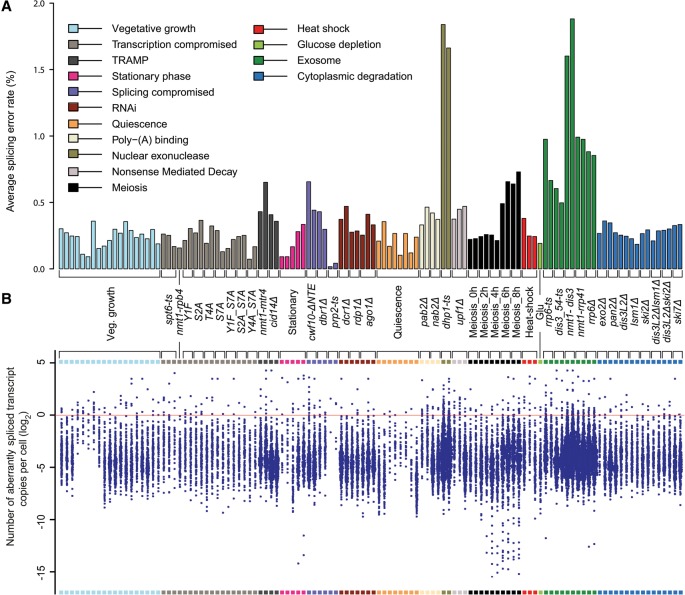
Splicing errors by exon skipping are eliminated by nuclear RNA surveillance. (*A*) Average splicing error for a given sample. Set of 2504 exon-skipping events for which both normal splicing and exon-skipping reads were identified in one or more samples. The splicing error rate for a locus is equal to corresponding local ESR (Supplemental Table S4). The values shown are averages of all local ESRs in a sample (Supplemental Table S7). Physiological conditions or mutants as indicated *below* were grouped and color coded according to cellular function or condition tested (Supplemental Table S1). (*B*) Number of aberrantly spliced transcripts per cell. To estimate this number, we multiplied local ESR for each exon-skipping event by corresponding transcript copy number (Supplemental Tables S8; Marguerat et al. 2012). Absolute cellular numbers of transcripts from growing cells were used as the gold standard for all samples, except for quiescence, where reported absolute numbers were used. Each blue point represents the copy number of an aberrantly spliced transcript. To avoid log_2_ of zero, exon-skipping events with local ESR/splicing error of zero were removed. Strains as indicated in *A*.

Assuming that exon-skipping events represent aberrantly spliced transcripts under the conditions tested here, the local ESR is a measure for the splicing error rate by exon skipping at a given locus. Using the 2504 exon-skipping events in the 1049 genes, where reads for both normal splicing and exon skipping were identified in at least one sample, we computed the mean of the local ESRs for all genes. These values thus estimate the average splicing error rate at a given locus under each condition (Fig. 4A; Supplemental Table S7).

In wild-type cells, only ∼0.24% of skipping errors were detectable, lower than the overall estimated splicing-error for human cells ∼0.7% (Pickrell et al. 2010). With compromised nuclear exonuclease activity (*dhp1-ts*), however, the mean splicing error rate by exon skipping increased greater than sevenfold (∼1.75%). Strains with compromised Dis3 function (*nmt1-dis3*) displayed a similar average splicing error (∼1.74%), whereas strains compromised for Rrp6 (*rrp6-ts* and *rrp6*Δ) showed a more moderate increase (∼0.84%). An increased splicing error rate by exon skipping was also observed during late meiotic differentiation (∼0.63%). Naturally, these are minimal estimates as other splicing mistakes will occur (e.g., errors in splice site selection), but also because the error rate might further increase in the simultaneous absence of several RNA surveillance pathways. We conclude that most splicing errors by exon skipping are masked by nuclear RNA surveillance.

### Exon-skipping transcripts are present at less than one copy per cell

Absolute cellular copy numbers for all *S. pombe* transcripts during vegetative growth and quiescence are available (Marguerat et al. 2012), based on transcriptome samples also analyzed here (Supplemental Table S1). Assuming that exon-skipping events represent splicing errors, we used these quantitative data, along with the local ESRs, to estimate cellular copy numbers for exon-skipping transcripts for each condition and splice variant (Supplemental Table S8).

Exon-skipping transcripts were largely present at less than one copy per cell, suggesting that these events occur only in a portion of cells in a population (Fig. 4B). Notably, although the diversity of exon-skipping events increased in nuclear RNA surveillance mutants and during late meiotic differentiation, the absolute numbers for any given event remained largely the same (Fig. 4B). This finding probably reflects that any given exon-skipping event is only present in a tiny fraction of the cell population, and compromised RNA surveillance will not substantially increase the population average of a specific splice variant but will increase the number of different events. Only 55 exon-skipping events were present at one or more copies per cell in one or more of 46 samples from different physiological conditions (Supplemental Table S9). However, even these higher numbers were variable among replicates and further increased in nuclear RNA surveillance mutants. We ranked these events based on their reproducibility across the 46 samples and identified only five exon-skipping events that exhibited one or more copies in at least five samples (Supplemental Table S9). We therefore conclude that exon-skipping events for any given transcript only occur in a small portion of cells in a population. This result further corroborates the view that exon-skipping events reflect splicing errors.

### Circular exonic RNAs are rare and stochastic

**Figure 5.**
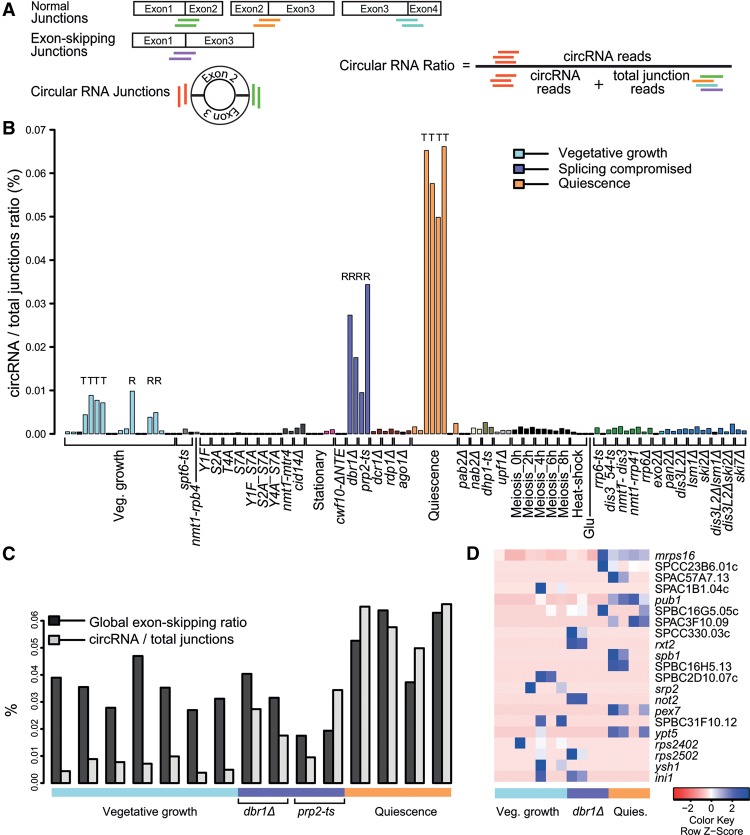
Analysis of circular exonic RNAs. (*A*) Scheme showing normal, exon-skipping, and circRNA transcripts. The colored lines *below* the junctions represent diagnostic reads for normal splice junctions (green, orange, cyan), exon-skipping junctions (purple), and circular junctions (red). These diagnostic reads were used to calculate global circular to total junction ratios and local circular to normal junction ratios used in *B*–*D*. (*B*) Global circular to total junction ratios across samples, reflecting the proportion of circRNA reads among total (normal and exon-skipping) junction reads. Physiological conditions or mutants as indicated *below* were grouped and color coded according to cellular function or condition tested (Supplemental Table S1). Samples were poly(A)-enriched, apart from those marked “T” (total RNA) or “R” (ribosomal depleted). (*C*) Global exon-skipping ratio compared to circular to total splice junction ratios in all non-poly(A)-enriched samples as indicated. (*D*) Heatmap for the 21 circRNAs most reproducible in total RNA and ribosomal-depleted samples, with corresponding genes indicated. The global circular to total junction ratio includes exon-skipping reads, whereas the local circular to normal junction ratio excludes exon-skipping reads in any given locus (Supplemental Table S11). Maximum distance between rows (circRNAs) was determined as indicated in Figure 3A.

Circular RNAs (circRNAs) have received recent attention owing to their regulatory roles; they are generated during splicing, either by circularization of a single exon or by joining the 3′-end of a downstream exon to the 5′-end of an upstream exon (Salzman et al. 2012; Ashwal-Fluss et al. 2014; Wang et al. 2014). Analogous to exon skipping, circRNAs can be readily identified by diagnostic RNA-seq reads (Fig. 5A; Salzman et al. 2012).

To analyze circRNAs in our data set, we constructed a database containing diagnostic junction sequences produced from all annotated single or reordered joint exons after all possible circularization events, only considering reads that perfectly matched to unique circRNA junctions. Only 895 diagnostic reads representing 254 different circRNAs from 223 genes were evident in our data (Supplemental Table S10). However, 16 circRNAs, accounting for ∼21% of diagnostic reads (188/895), originated from genes without any annotated introns and might therefore reflect baseline noise. The putative circRNAs included 40 of the 44 circRNAs identified by Wang et al. (2014); they were only supported by very few reads, with ∼40% being predicted by just one read (Supplemental Table S10). About 66% of diagnostic reads (588/895) originated from a single circularized exon, and the number of reads decreased with increasing numbers of introns between the joint exons (Supplemental Fig. S7). About 94% of circRNAs (238/254) originated from genes containing three or more exons (Supplemental Fig. S8), in line with reports that flanking introns facilitate circularization (Jeck et al. 2013; Ivanov et al. 2014; Liang and Wilusz 2014; Zhang et al. 2014).

Given that circRNAs do not contain poly(A) tails, the low diagnostic read counts could be explained by most of our RNA samples being poly(A)-enriched before sequencing. Indeed, higher diagnostic read counts were evident in the 15 total RNA and ribosomal RNA-depleted samples compared to the poly(A)-enriched samples (Fig. 5B). Even for these samples not enriched for poly(A) RNAs, however, the ratios of circular to total junction reads were smaller than (in vegetative growth) or similar to (in quiescence and splicing mutants) the global ESR (Fig. 5C). To obtain a more conservative set, we computed the local circular to normal junction ratios for circRNAs detected by one or more reads in two or more biological replicates during growth, quiescence, and in *dbr1*Δ debranching mutant cells. This approach retained 21 circRNAs, including the four circRNAs shown to be RNase R resistant (Wang et al. 2014). The circular to normal junction read ratios were generally low (mean ∼3%), albeit a few circRNAs became consistently more stable during quiescence and in *dbr1*Δ cells (Fig. 5D; Supplemental Table S11). We conclude that most circRNAs reflect biological, experimental, or bioinformatics noise, although a few might become stabilized during quiescence and in splicing-deficient cells.

### Widespread cryptic splice sites and novel introns

Exon skipping represents only one mechanism by which alternative splice variants could be generated; additional mechanisms are intron retention and alternative 5′- and 3′-splice donor and acceptor sites. Recent studies reported numerous novel introns in budding yeast (Volanakis et al. 2013; Kawashima et al. 2014) and fission yeast (Awan et al. 2013; DeGennaro et al. 2013). We therefore analyzed our data set for cryptic (rarely used) splice sites and novel (efficiently spliced) introns. To this end, we developed an exhaustive search algorithm to partition the genome based on all possible splice donor and acceptor dinucleotide combinations rather than considering only the consensus 5′-(GU) and 3′-(AG) splice sites (Supplemental Fig. S9). We thus generated a database containing all junctions that bridge all possible known or unannotated introns. Against this database, we then aligned all RNA-seq reads that could not be mapped to the genome or annotated transcriptome. A clear enrichment for consensus 5′- and 3′-splice signals was evident (∼98% of unannotated events), indicating that these unannotated junctions reflect spliceosome-mediated splicing (Supplemental Fig. S10). About 2% of these junctions were associated with noncanonical sequences (Supplemental Table S12), and ∼5% of all unannotated junctions were expected to be false positives. Considering only consensus 5′- and 3′-splice signals, we identified a total of 5720 unannotated splicing events supported by 1,165,427 unique junction reads. We identified ∼52% (115/222) of the novel introns reported by Awan et al. (2013) and 307 known introns (Supplemental Table S12), which were picked up because some mRNA transcripts were misannotated or omitted from the Ensembl transcriptome used (release 13) (Flicek et al. 2014); their identification therefore provided a positive control to corroborate our approach.

The 5720 unannotated introns were distributed predominantly within protein-coding genes (∼61%), but also in noncoding RNAs and intergenic regions (Supplemental Fig. S11). Interestingly, 967 protein-coding genes with unannotated introns have not been known to be spliced. The unannotated introns displayed a similar length distribution to the 5361 annotated introns, with a marginal tendency to be longer (Supplemental Fig. S12). They also had near identical splice-signal consensus sequences (Supplemental Fig. S13), which were located in comparable distances relative to the 3′-ends of introns. Unannotated introns, however, exhibited higher GC content than annotated introns (Supplemental Fig. S14). For 1910 of the unannotated introns, one cryptic or novel site was spliced to an annotated site (thus altering the size of a known intron), whereas 3810 of the unannotated introns were derived from two cryptic or novel splice sites (Supplemental Table S12).

**Figure 6.**
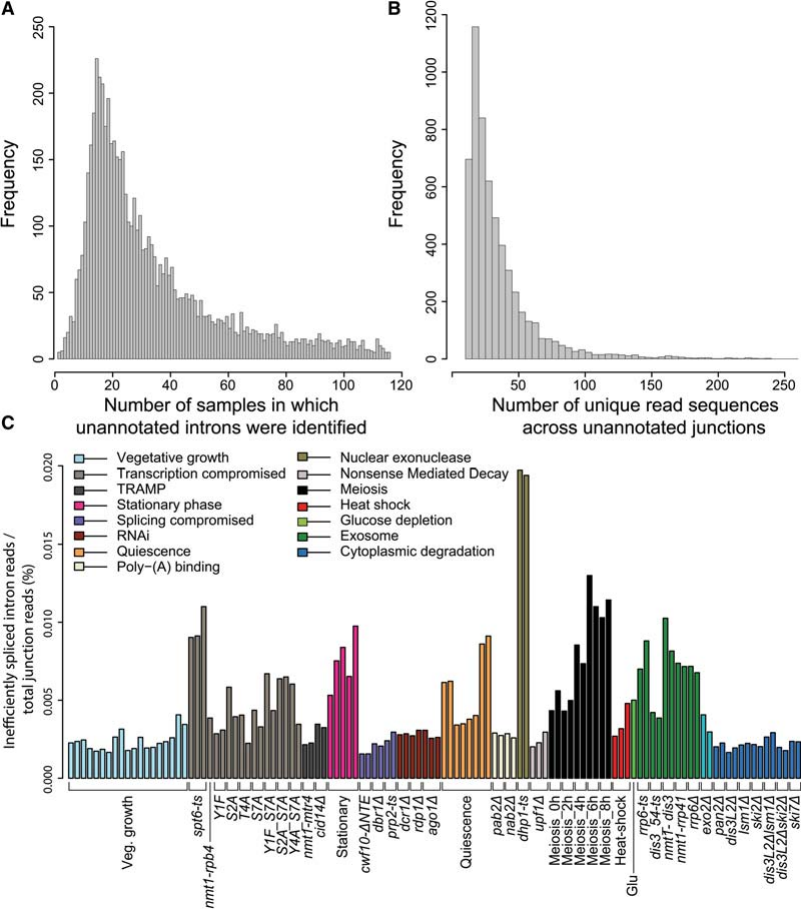
Distribution of novel introns with respect to samples in which they were identified (*A*) and the sum of their supporting unique read sequences across the unannotated splice junctions (*B*). A total of 5720 unannotated splicing events are shown. (*C*) Inefficiently spliced introns accumulate in nuclear surveillance and transcription mutants, meiotic differentiation, and during stationary phase and quiescence. A cutoff of 70 or fewer unique sequences across junctions was applied, resulting in ∼92% (5235/5720) of least efficiently spliced introns (for values in each sample, see Supplemental Table S12). We crudely separated potentially novel introns from cryptic introns by applying an arbitrary threshold (more than 70 unique sequences across the junction; 485/5720 putative novel introns). Ratios of sample-specific inefficiently spliced intron reads to total junction reads are shown, reflecting the proportion of exon–exon reads of inefficiently spliced introns among total exon–exon junction reads. Physiological conditions or mutants as indicated *below* were grouped and color coded according to cellular function or condition tested (Supplemental Table S1).

Skewed distributions were evident for the number of samples containing unannotated introns (Fig. 6A) and for the number of unannotated junction read sequences (Fig. 6B). A high correlation was evident between the number of unannotated introns and sequencing depth, implying that most of these splicing events are rare and aberrant (Supplemental Fig. S15). As for exon skipping, many of the unannotated introns tended to accumulate in mutants with defective nuclear degradation and also in the *spt6* mutant with compromised transcription regulation (Supplemental Fig. S15, indicated by arrows). The latter result is in agreement with findings by DeGennaro et al. (2013). Applying a conservative cutoff, we further analyzed ∼92% (5235/5720) of the unannotated introns that were least efficiently spliced, revealing a signature across the biological conditions; these cryptic splicing events accumulated in nuclear degradation and some transcription mutants, during late meiosis, and in stationary and quiescent cells (Fig. 6C).

We tested 30 unannotated introns by RT-PCR: 29/30 displayed the expected size, and 25/30 were also validated by Sanger sequencing (Supplemental Fig. S16; Supplemental Table S13). Expression profiles of selected genomic regions highlight the differences between novel introns missed by current annotation and cryptic introns likely to represent splicing noise (Supplemental Fig. S17). As for annotated introns, many unannotated introns showed higher expression in *dbr1*Δ mutant cells, whereas the expression of known exons remained unchanged; this increase was biased by intron length, a signature indicative of lariats (Supplemental Fig. S18; Bitton et al. 2014). These observations support the conclusion that these unannotated introns are spliced by the cellular spliceosome rather than reflecting experimental or bioinformatics noise.

Taken together, most unannotated introns only appeared in a small number of samples and were supported by low numbers of junction reads, and these events likely reflect inefficiently and erroneously spliced introns. Like exon-skipping transcripts, transcripts containing such cryptic introns were targeted by the nuclear exosome and accumulate in specific physiological conditions. Introns identified in most of the samples and supported by a high number of reads were much rarer; these introns are likely bona fide, novel introns that have been missed by prediction algorithms. These findings reveal that splicing of the fission yeast transcriptome is much more pervasive than appreciated, but most of these unannotated events might reflect splicing errors without cellular function.

## Discussion

Splicing is pivotal for gene expression, and any mistakes can be detrimental. Alternative splicing has been considered mainly as a mechanism to diversify proteomes, with >90% of human genes potentially producing multiple transcripts from a single locus (Wang and Burge 2008; Wang et al. 2008). Here we present data highlighting that many alternative splice variants in fission yeast are not necessarily functional. Using a systematic analysis of RNA-seq data, we conclude that widespread but lowly expressed exon-skipping events and inefficiently spliced introns reflect splicing errors that are actively degraded in the nucleus. These results raise the possibility that alternative splicing has originally emerged as a by-product of cellular splicing errors and has then been coopted during evolution for specialized protein functions in complex eukaryotes. This hypothesis is supported by two recent studies showing that alternative splicing signatures are species-specific and differ significantly even between closely related species (Barbosa-Morais et al. 2012; Merkin et al. 2012).

We have previously shown that exon skipping is not a consequence of bioinformatics noise by validating a subset of exon-skipping events using gene-specific RT-PCR and identifying 82 exon-skipping lariat reads in debranching deficient *dbr1*Δ cells (Bitton et al. 2014). The coherent increase of exon-skipping events in specific sets of biological samples also indicates a biological origin. The following pieces of evidence, however, indicate that exon-skipping transcripts in fission yeast represent splicing errors that may be without function. First, exon skipping is a rare and erratic phenomenon, supported by only a few RNA-seq reads compared to reads supporting normal splicing. Second, exon-skipping events are randomly scattered throughout the transcriptome and show no obvious dependency on intron or exon length, on expression levels, or on physiological conditions (except during late meiosis); however, they are positively correlated with the number of splicing reactions a given gene undergoes, in line with findings from human cells (Pickrell et al. 2010). Third, exon-skipping transcripts are not only much less abundant compared to their normal counterparts, but also accumulate in cells with impaired nuclear RNA surveillance and during late meiosis, when RNA surveillance might be down-regulated. Fourth, exon-skipping transcripts are present at less than one RNA copy per cell on average in a population; such low levels are diagnostic for actively repressed transcripts that have no function under the given condition (Marguerat et al. 2012). Fifth, exon-skipping events show no enrichment above random to preserve the open reading frames, appear not to be associated with translating ribosomes, and are therefore unlikely to be translated. Nevertheless, we cannot exclude that some of the exon-skipping events are functionally relevant during specialized conditions not investigated here.

In budding yeast, Rat1/Dhp1 and the nuclear exosome are involved in degrading four exon-skipping transcripts (Egecioglu et al. 2012). Here we show that exon-skipping events are degraded globally by the same two surveillance pathways in the distantly related fission yeast. It is not clear how these two pathways can recognize aberrantly spliced transcripts. Available data are indicative of a strong competition between RNA splicing and mRNA decay (Lemieux et al. 2011). Accordingly, we speculate that exon skipping is less efficient than normal splicing, and the extended kinetics could be outcompeted by mRNA decay reactions. We also observed a relatively low, but significant, accumulation of exon-skipping transcripts in cells with mutations in the NMD pathway. These data suggest that several surveillance mechanisms can recognize erroneous exon skipping and back one another up (Sayani and Chanfreau 2012), although the nuclear exosome and Rat1/Dhp1 play dominant roles. It is surprising that NMD seems to play only a minor role in degradation of aberrantly spliced transcripts. Inefficient splicing in budding yeast could modulate gene expression via NMD (Kawashima et al. 2014) or SMD (Volanakis et al. 2013). In mammalian cells, widespread intron retention also tunes gene expression (Braunschweig et al. 2014), and thousands of introns are spliced at low efficiency (“detained introns”), which are not subjected to NMD but do affect gene expression levels (Boutz et al. 2015). In our data, relative changes in abundance of specific splice variants between mutant and wild-type samples were not accompanied by changes in corresponding transcript levels. We therefore conclude that exon skipping is not widely used to modulate gene expression in fission yeast.

Exon-skipping transcripts increased during late stages of meiotic differentiation, but our data do not support any functional role of exon skipping during meiosis. Even the increased levels of exon-skipping transcripts are still at low abundance relative to their normally spliced counterparts. Nuclear-exosome and *dhp1* transcripts decreased during sexual differentiation, although it is not clear whether this reflects down-regulation of RNA surveillance during meiosis. It has been reported that Rrp6 is degraded during meiosis in budding yeast, which plays a regulatory role for meiotic progression (Lardenois et al. 2011). However, a recent study has shown that the exosome remains active during budding yeast meiosis (Frenk et al. 2014).

Recent findings from multicellular eukaryotes indicate that exonic circRNAs are common, functional, and can compete with normal splicing (Salzman et al. 2012; Hansen et al. 2013; Memczak et al. 2013; Ashwal-Fluss et al. 2014). In fission yeast, exonic circRNAs were augmented in the non-poly(A)-enriched samples as expected, in contrast to the exon-skipping transcripts, indicating that the latter are polyadenylated. Analogous to exon skipping, the very low diagnostic read counts and largely incoherent biological signature across conditions suggest that most circRNAs are not functional in fission yeast, although a few accumulate during quiescence and in splicing-deficient cells. It is possible, however, that we underestimate circRNA occurrence because the sequence library preparation was not optimized for such RNAs (Lasda and Parker 2014).

Our exhaustive search for cryptic splice sites and novel introns has doubled the number of splicing events in this well-studied model organism. Although the minority of efficiently spliced novel introns requires an update of genome annotations, the many cryptically spliced introns probably reflect splicing errors. Ribosome profiling in *S. pombe* has suggested only a single case of intron retention and several examples of incorrect annotation (Duncan and Mata 2014), supporting the view that cryptic splicing events mostly represent errors. Accordingly, these cryptic events accumulate in nuclear-exosome mutants as do exon-skipping transcripts. These results further highlight that splicing errors are far more common than previously recognized, and they are targeted by the nuclear exosome. Similar phenomena have been documented in human cells (Melamud and Moult 2009; Pickrell et al. 2010). Cryptically spliced RNAs also accumulated when the transcriptional machinery was compromised, most notably in *spt6* mutant cells, highlighting the tight coordination between transcription and splicing. Cryptically spliced RNAs also slightly increased under certain physiological conditions, and it is possible (although unlikely given their low expression) that some of them might have specialized roles in these conditions.

Our analyses, together with observations made by other studies (Melamud and Moult 2009; Pickrell et al. 2010), refine the dogma that alternative splicing in eukaryotes is limited to the production of functionally relevant transcripts. Even in human cells, a considerable fraction of alternative splice variants could simply reflect splicing errors, which in turn are actively targeted by RNA surveillance mechanisms.

## Methods

### Strain list, RNA isolation, and sequencing

The genetic background, growth conditions, type of RNA-seq [i.e., poly(A)-enriched, total RNA, or ribosomal depletion], and the sources of all *S. pombe* strains used in this study are specified in Supplemental Table S1. For poly(A)-enriched libraries, RNA extraction, library preparation, and sequencing protocols were as described by Lemieux et al. (2011), for total RNA as described by Marguerat et al. (2012), and for ribosomal-depleted libraries as described by Bitton et al. (2014). Publicly available data sets were downloaded from GEO, SRA, or ArrayExpress repositories (accessions indicated in Supplemental Table S1). Because the exon-skipping discovery pipeline does not support paired-end reads, only the 5′-end of the read was used for all paired-end data sets (Livesay et al. 2013; Schwer et al. 2014). An earlier version of our intron discovery pipeline supported paired-end data sets; thus, both ends of the paired-end reads from Rhind et al. (2011) were used for novel intron discovery.

### Genome level alignments and annotation

Sequence reads of “X” base length (see Supplemental Table S1 for exact read length of each RNA-seq experiment) originating from each sample were aligned using Bowtie 0.12.7 (Langmead et al. 2009) to the *S. pombe* genome sequence (Ensembl *S. pombe*, Build EF1, release 13) (Flicek et al. 2014) and to the corresponding exon–exon junctions database. For exon-skipping analysis, up to 3 base pair (bp) mismatches were allowed. Reads that matched multiple loci were removed from further analysis, and the resultant alignment files were processed to generate “pile-ups” against each chromosome (for total number of mappable reads in each sample, see Supplemental Table S1). Importantly, throughout our exon-skipping analysis, we used only consecutive (normal) and nonconsecutive exon–exon junction reads (Fig. 1A) and reads that span the exon-intron boundaries (Fig. 1A), while the remaining reads were ignored. Furthermore, reads that could not be mapped to the genome or transcriptome with three mismatches were used for novel spliced region analysis as well as for circRNAs; whereas in both analyses, only unique alignments without mismatches were considered. Since global and local exon-skipping ratios are compared between samples, no normalization is required for these relative data that are internally normalized. However, for the correlations of exon skipping with exon/intron length or with gene expression (Supplemental Figs. S2–S4) and for expression profiles of unannotated introns in *dbr1*Δ cells (Supplemental Fig. S18), normalized expression levels were calculated as described in figure legends and by Bitton et al. (2014). Normalization and differential expression analysis for the meiotic data set were carried out using the DESeq package (Anders and Huber 2010) as described (Bitton et al. 2014).

### Exon–exon, exon-skipping, and circular junction databases

Searches were performed against the genome sequence combined with a database of all possible exon–exon junction sequences that could be generated from Ensembl *S. pombe* annotation (release 13). To ensure that an “X”-base read is mapped to a splice junction (see Supplemental Table S1 for exact read lengths), only the last (“X”-6) bases of the first exon and the first (“X”-6) bases of the second exon were considered (if the exon exceeded length “X”-6). In this way, reads that overlapped a junction by <6 nucleotides (nt) were excluded. Reads that matched to more than one junction or elsewhere in the genome were also discarded. For the exon-skipping database, all possible exon-skipping junctions were combined with all normal exon–exon junctions into a single database. To allow competition between all putative transcript sequences, we simultaneously aligned all sequence reads against the junction database attached to the reference genome, retaining only reads mapping to a single location, with up to three mismatches. The exon-skipping database used for mapping RPFs was constructed as described above with read length set to 25 bp, given that the protected fragments considered were 28–30 bp in length. For the circular junction database used to identify circRNAs, all possible exon–exon junctions of shuffled exons within a given gene were combined together with circular junctions from single exons (tail to head junctions), including those that only contain one exon (negative control). To maximize mapping of reads while accounting for varying read lengths in our data set (49–76 bp), the read length was set to 57 bp.

### Analysis of ribosome profiling data

Ribosome profiling data (Duncan and Mata 2014) were downloaded from ArrayExpress (accession E-MTAB-2176 for vegetative haploid cells; E-MTAB-2179 for *pat1-114* meiosis, replicate 1; and E-MTAB-2265 for *pat1-114* meiosis, replicate 2). Reads were trimmed to remove barcodes (4 bp) and unique molecular identifiers (5 bp). Using Bowtie, reads were initially aligned to the *S. pombe* genome combined with the exon-junction database. Only normal exon–exon and exon-skipping reads of 28–30 bp length that were mapped uniquely and with no mismatches were considered. Global and local exon skipping to normal RPF ratios were calculated as for the RNA-seq data (Supplemental Table S6).

### Cryptic splice site and novel intron analysis

To identify cryptic splice sites and novel introns independently of prior splice-site knowledge, we first divided the fission yeast genome into six batches (5′-3′ direction), based on all annotated genes, as follows. In each batch, we stored a fraction of the genome containing different gene sequences ±300 bp upstream and downstream to ensure identification of introns within untranslated regions (UTRs) and any intervening sequences (i.e., intergenic regions). Thereafter, we partitioned each region within each batch based on all possible splice donor and acceptor dinucleotide combinations (i.e., 4^2^ × 4^2^ = 256 combinations) (Supplemental Fig. S9). By doing so, we generated a database containing all possible introns (minimum size of 10 bp) around which splice junctions were constructed as described below. A 10-bp cutoff was used to enable identification of any tiny introns. Our data set included RNA-seq reads of varying lengths (49–76 bp); to maximize read mapping, we created junctions around putative introns using 57 bp upstream of and 57 bp downstream from their flanking sequences (or entire flanking sequence if shorter than 57 bp). Thereafter, all junctions produced from a given splice donor and acceptor combination were stored as a single database for each batch. Then, using Bowtie, we aligned to each database all RNA-seq reads that could not be mapped to the genome or the annotated transcriptome (33 samples), but this time we retained only unique matches without tolerating any mismatches. The remaining 83 samples were aligned only to databases produced from the consensus 5′-(GU) and 3′-(AG) splice donor and acceptor sites. Thereafter, read alignments from all databases, dinucleotide combinations, and samples were combined into a single file. Using customized Perl and R scripts, we filtered out nonunique matches or those from rRNA regions in both orientations as well as reads with an overhang of <6 bp across the junction. To further minimize false positive mappings, we calculated the false discovery rate (FDR) based on results obtained from alignments of 33 samples that were searched against all possible dinucleotide combinations. To do so, we considered all combinations other than 5′-GU and 3′-AG splice donor and acceptor sites as random matches. Then, we calculated the FDR as follows: FDR = (number of random junctions) / (number of 5′-GU and 3′-AG junctions + random junctions), while the number of unique sequence reads starting at different locations along the junction was used as the varying cutoff. FDR analysis revealed that only ≤5% random matches were retained in the reported list if a given junction was supported by more than 13 unique sequences across 33 samples. This threshold was then applied to junctions obtained from all 116 transcriptomes. Under this threshold, we identified 6238 putative canonical junctions (5′-GU–3′-AG junctions only) and 105 noncanonical splice sites. However, due to the ±300 bp upstream and downstream gene flanking sequences used for our database, there was a low degree of redundancy. Therefore, 5899 novel canonical splicing events led to the identification of 5720 novel introns (supported by 1,165,427 unique junction reads), while the remaining 339 events corresponded to 307 known introns (supported by 657,527 unique junction reads). The 105 noncanonical junctions (supported by 17,564 reads) led to the identification of 104 putative noncanonical introns. By comparing the unannotated splice junctions against the annotated introns, we identified 1910 introns that were based on one annotated and one unannotated splice site (1155 with an annotated 5′-splice donor) and 1007 with an annotated 3′-splice acceptor, while 252 introns were in common (i.e., served as cryptic donor/acceptor to two different annotated introns).

### Comparison of novel and unannotated splicing events

The GC content of both the unannotated splicing events and annotated intron sets was determined using the “geecee” function within the European Molecular Biology Open Software Suite (EMBOSS) (Rice et al. 2000). Branch-site predictions were performed using FELINES (Drabenstot et al. 2003). Two FASTA files containing 5686 novel spliced regions and 5283 annotated introns with canonical splice donor and acceptor sites (5′-GU and 3′-AG, respectively) and of length >20 bp were analyzed using FELINES with default settings. Consensus branch-site, 5′ and 3′ sequence logos were plotted using WebLogo (Crooks et al. 2004).

## Data access

RNA-sequencing data from this study have been submitted to the European Nucleotide Archive (ENA; http://www.ebi.ac.uk/ena) under accession number PRJEB7379.

## Supplementary Material

Supplemental Material
