# Supplementary material for: Widespread exon skipping triggers degradation by nuclear RNA surveillance in fission yeast
Source: Genome Res. 2015 Jun;25(6):884–96. doi: 10.1101/gr.185371.114 (PMC4448684; doi:10.1101/gr.185371.114)
Supplement: Supplemental Material [file supp_25_6_884__index.html]

Widespread exon-skipping triggers degradation by nuclear RNA surveillance in fission yeast — Widespread exon skipping triggers degradation by nuclear RNA surveillance in fission yeast — Widespread exon skipping triggers degradation by nuclear RNA surveillance in fission yeast — Supplemental Material 

# Widespread exon skipping triggers degradation by nuclear RNA surveillance in fission yeast

## Supplemental Material

**Files in this Data Supplement:**

- Supplemental Material.pdf
- Supplemental Tables.xlsx
