# Supplementary material for: Widespread exon skipping triggers degradation by nuclear RNA surveillance in fission yeast
Source: Genome Res. 2015 Jun;25(6):884–96. doi: 10.1101/gr.185371.114 (PMC4448684; doi:10.1101/gr.185371.114)
Supplement: Supplemental Material [file supp_gr.185371.114_Supplemental_Material.pdf]

Widespread exon-skipping triggers degradation by nuclear RNA surveillance in fission yeast

Danny Asher Bitton, Sophie Radha Atkinson, Charalampos Rallis, Graeme Christopher Smith, David Andrew Ellis, Yuan Yi Constance Chen, Michal Malecki, Sandra Codlin, Jean-François Lemay, Cristina Cotobal, François Bachand, Samuel Marguerat, Juan Mata, and Jürg Bähler

Supplemental Material

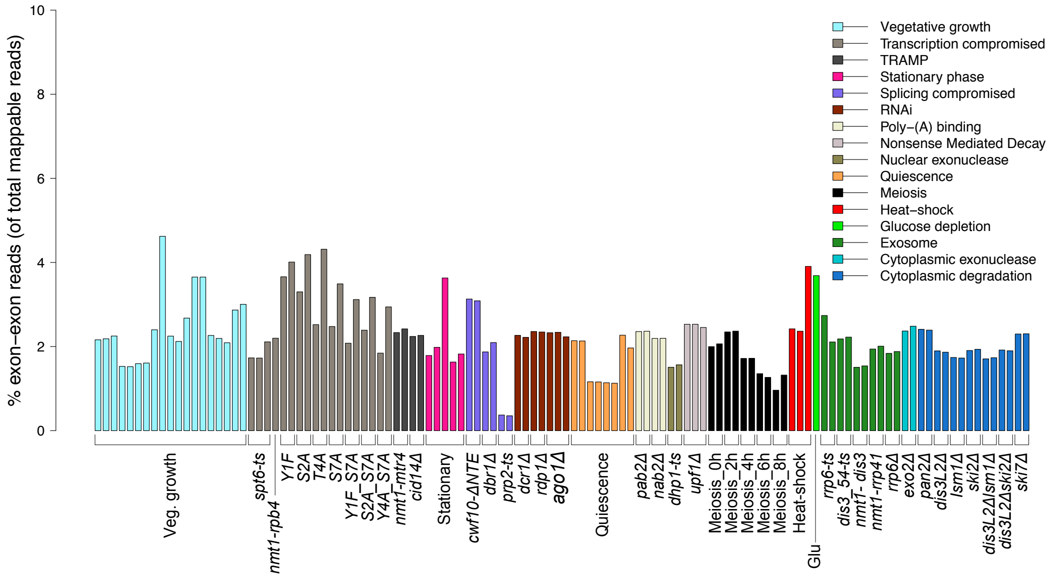

Supplemental Figure S1. Fractions of exon-exon junction reads (%) among total mappable RNA-seq reads in 116 transcriptomes interrogated here. Physiological conditions or mutants as indicated below were grouped and color-coded according to cellular function or condition tested. For full description of each strain, see Supplemental Table S1.

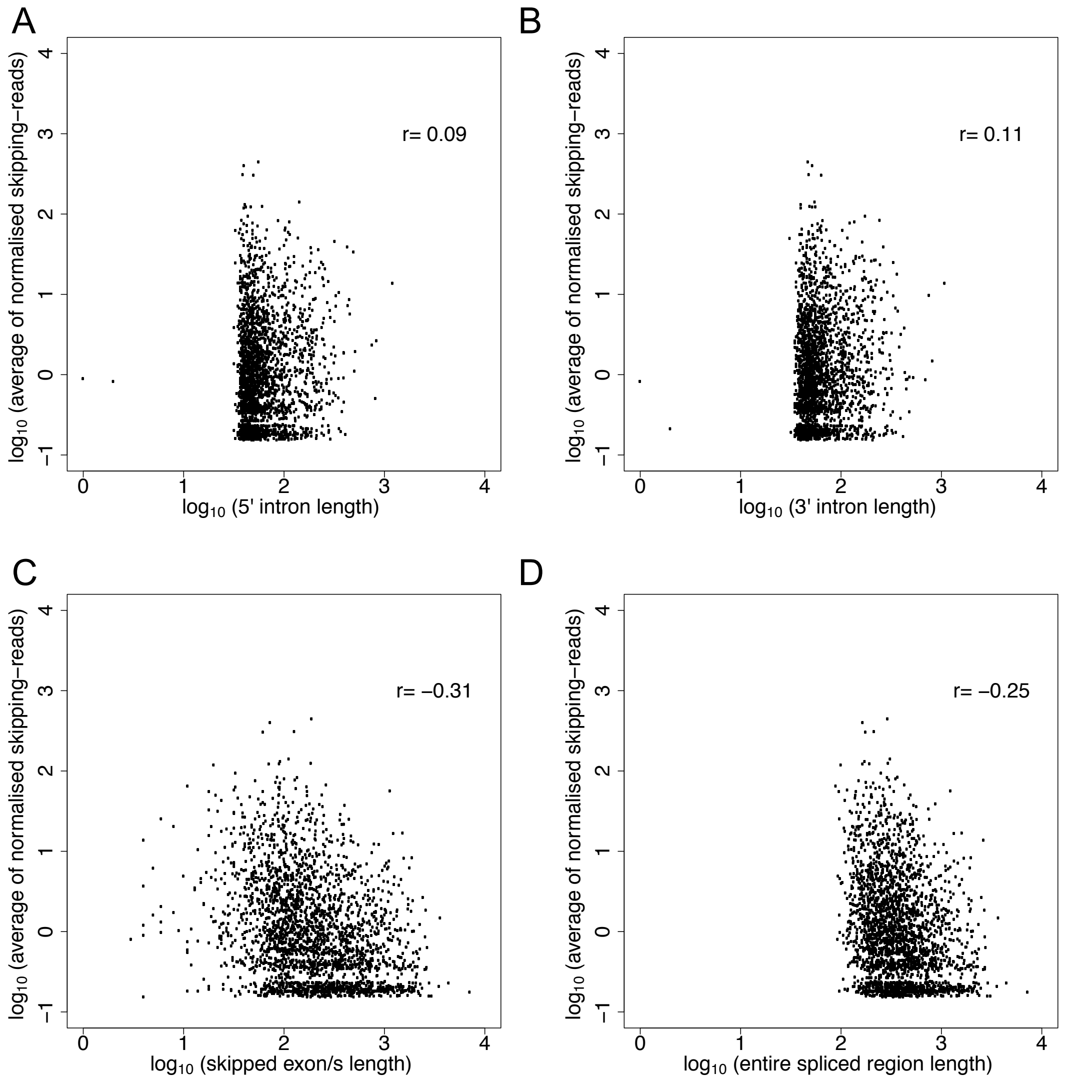

Supplemental Figure S2. Average abundance of exon-skipping events is not substantially correlated with (**A**) 5'-intron size; (**B**) 3'-intron size; (**C**) skipped exon length; or (**D**) the length of entire spliced region. Each dot represent an exon-skipping event (total of 2,574), with the average of normalized exon-skipping reads summarised across 116 transcriptomes ('Y' axis; exon-skipping reads were normalised by the total number of reads in a given sample and resultants ratios were multiplied by a constant 10^9). The Pearson's correlation coefficients 'r' are indicated.

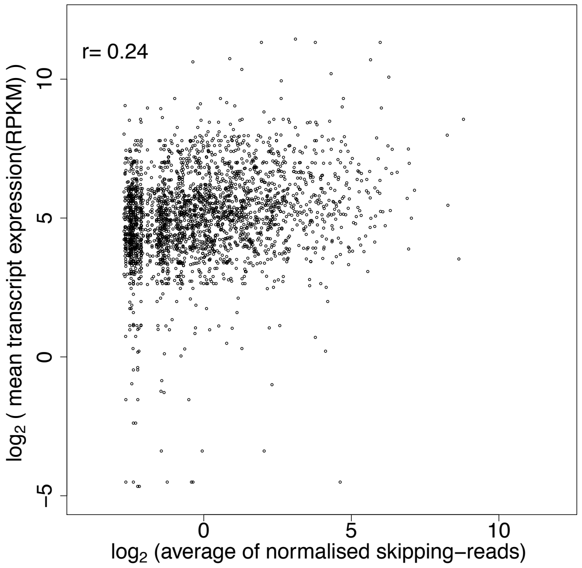

Supplemental Figure S3. Average abundance of exon-skipping events is not substantially correlated with expression of corresponding transcripts. Each point represents an exon-skipping event (total: 2,574 events) with the average of normalised exon-skipping reads summarised across 116 transcriptomes (exon-skipping reads were normalised by the total number of reads in a given sample and resultants ratios were multiplied by a constant 10^9). The mean RPKM (Reads Per Kilo base per Million) levels and number of reads were summarised across 116 transcriptomes (‘X’ and ‘Y’ axes, respectively). The Pearson’s correlation coefficient ‘r’ is indicated.

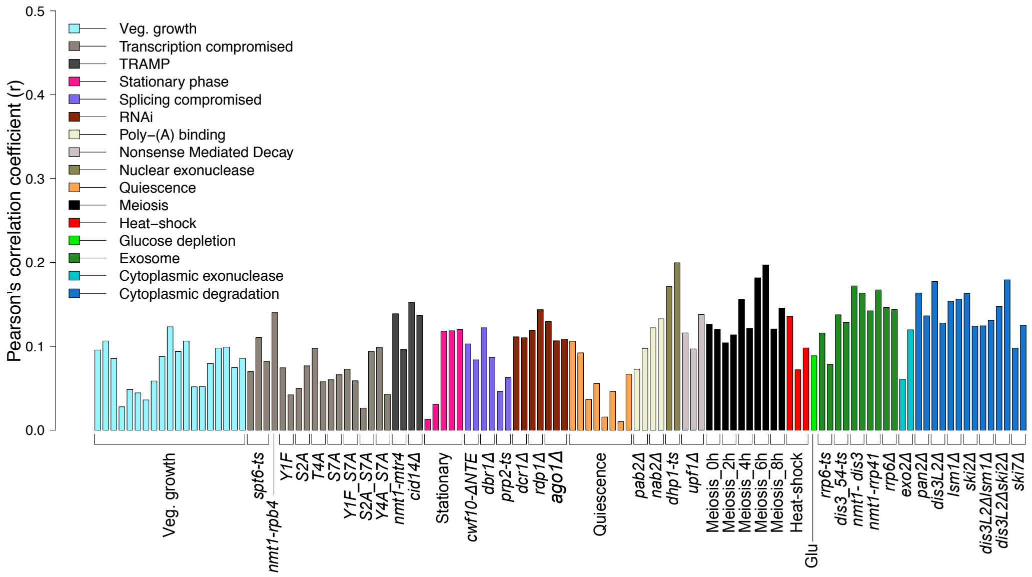

Supplemental Figure S4. Abundance of exon-skipping events is not correlated with expression of corresponding transcripts at any of the 116 samples interrogated in this study. For each sample, the normalized exon-skipping reads of all 2,574 exon-skipping events was tested against their corresponding transcript RPKM (Reads Per Kilo base per Million) levels. Exon-skipping reads were normalised by the total number of reads in a given sample and resultant ratios were multiplied by a constant 10^9. Sample specific Pearson’s correlation coefficient ‘r’ are plotted (‘Y’ axis). Physiological conditions or mutants as indicated below were grouped and color-coded according to cellular function or condition tested. For full description of each strain, see Supplemental Table S1.

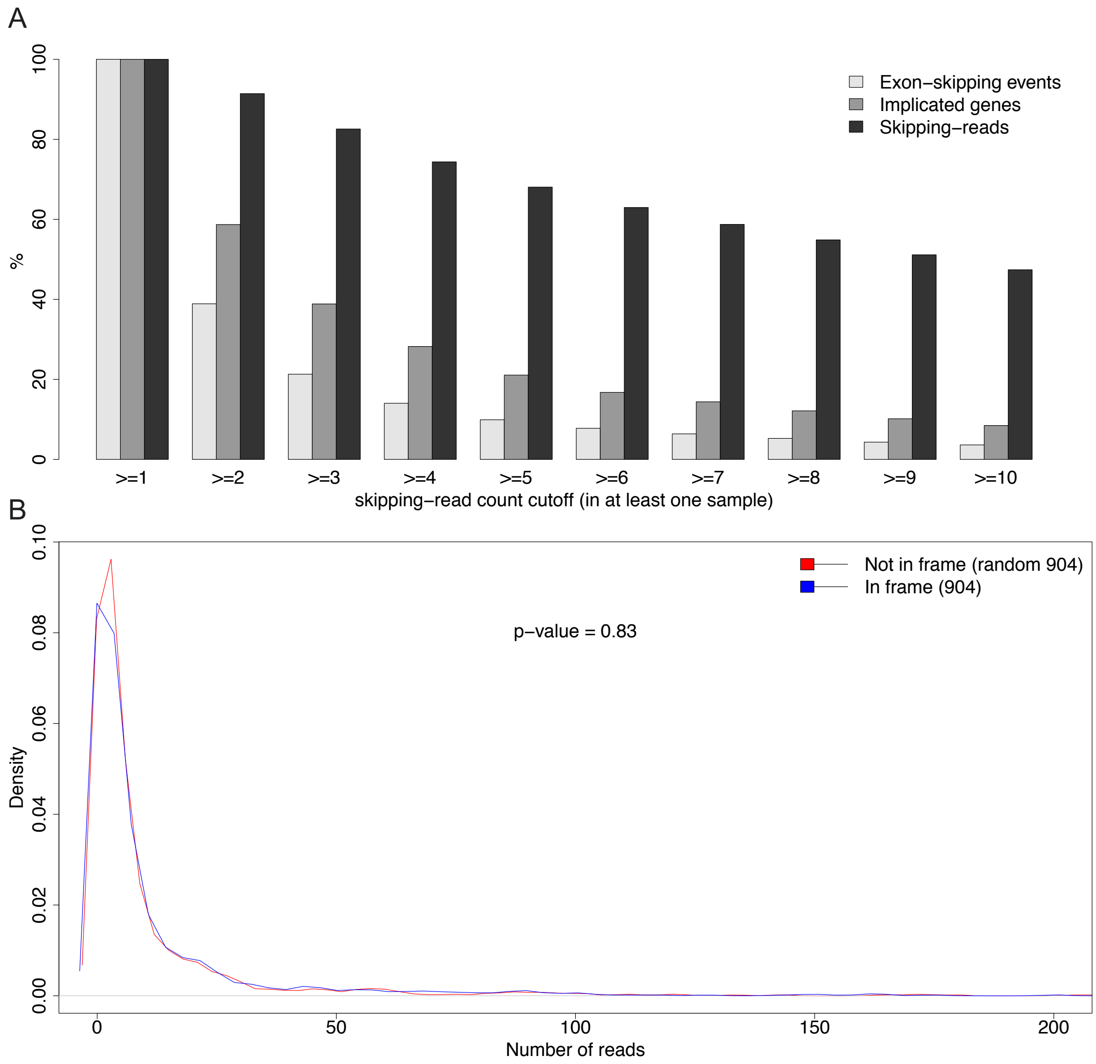

Supplemental Figure S5. Defining high-confidence exon-skipping set and assessing the read distribution of exon-skipping events that maintain open reading frame.
(A) Proportions of exon-skipping events, their host genes as well as corresponding exon-skipping reads as a function of a skipping-read cutoffs. A cutoff of ≥9 reads was used to derive the high-confidence set of 111 exon-skipping events originated from 108 genes and supported by 22,817 exon-skipping reads among the 44,616 reads identified here.
(**B**) Only 904 of the 2,574 skipped exons were divisible by 3, and they showed no significant increase in the number of skipping-reads (Wilcoxon Rank Sum test; p value as indicated) compared to a random set of 904 exon-skipping events that did not maintain the frame.

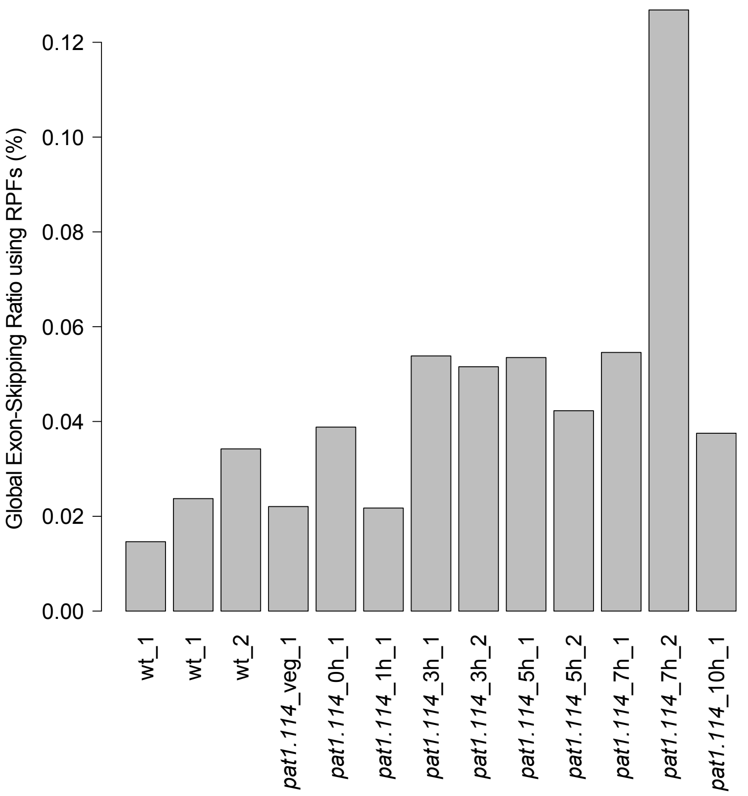

Supplemental Figure S6. Global exon-skipping to normal splice junction RPF ratios. Ribosome footprint data obtained from Duncan and Mata (2014). RPFs – Ribosome protected mRNA fragments. Reads were aligned using Bowtie 0.12.7, only considering 28-30 bp RPFs that mapped uniquely to the junction database with no mismatches. Total number of diagnostic skipping RPFs was divided by total junction RPFs (i.e. exon-skipping plus normal exon-exon RPFs).

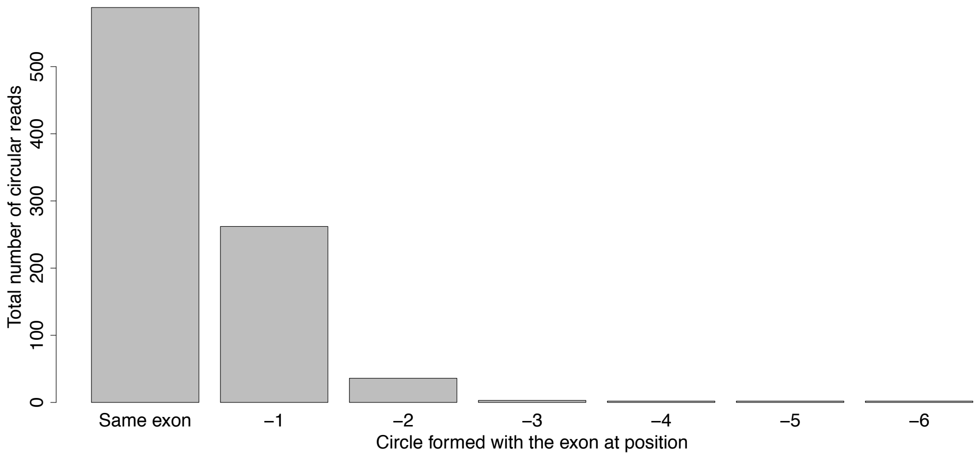

Supplemental Figure S7. CircRNA transcripts are mostly formed by circularization of a single exon. The X-axis refers to position of upstream exon which pairs with a given downstream exon, with -1 being the immediate upstream exon, -2 the second upstream exon, etc. Reads that initially failed to map to the *S. pombe* genome and transcriptome were aligned using Bowtie 0.12.7. Only those that were mapped uniquely to the circular junction database with no mismatches were considered (895 in total).

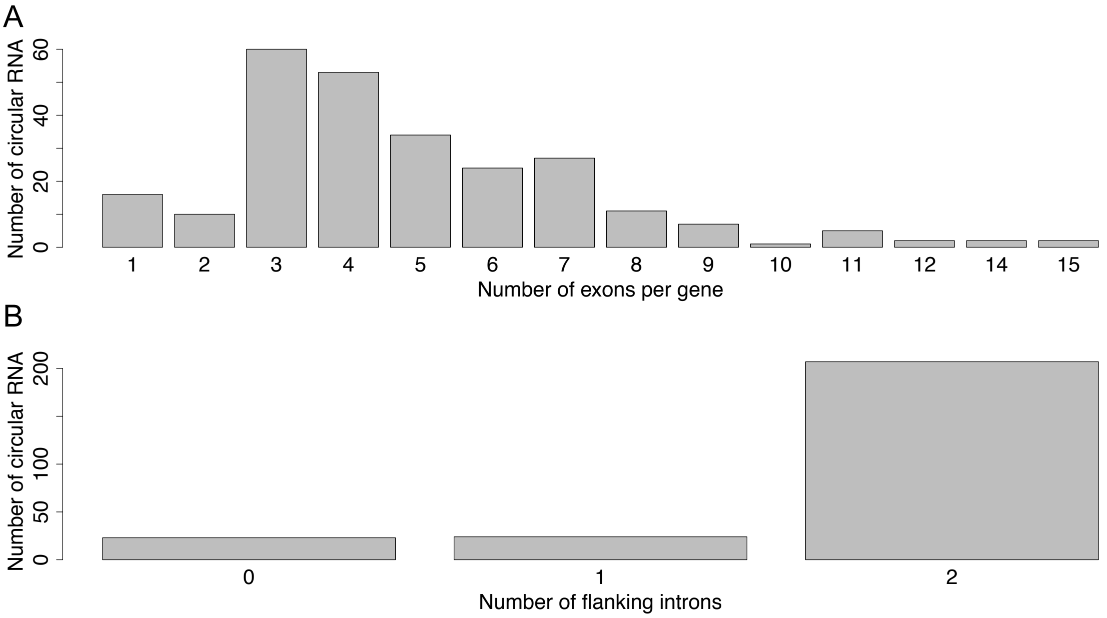

Supplemental Figure S8. Exonic circRNAs tend to form in genes having 2 introns or more. (**A**) CircRNAs were binned according to number of exons contained in host gene.
(**B**) CircRNAs were binned based on number of annotated introns flanking their 5’ and 3’ ends. Reads that initially failed to map to *S. pombe* genome and transcriptome were aligned using Bowtie 0.12.7. Only reads that mapped uniquely to circular junction database with no mismatches were considered.

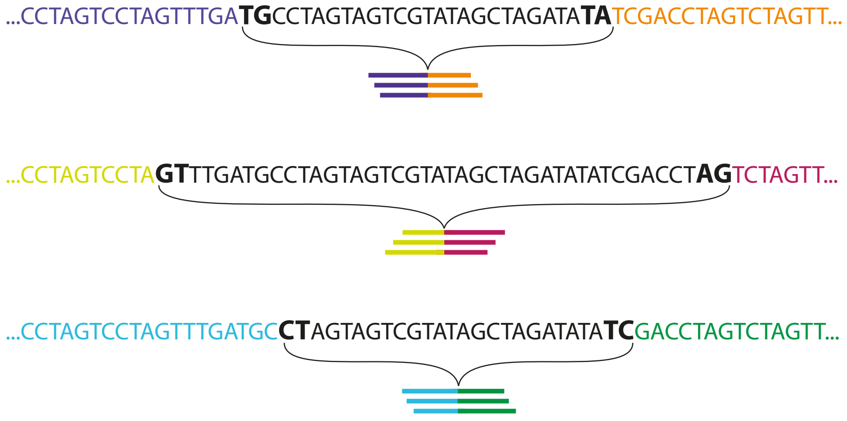

Supplemental Figure S9. Cartoon illustrating exhaustive search algorithm for discovery of novel introns and cryptic splice sites. In brief, the fission yeast genome was divided into six batches (5’-3’ direction) based on all annotated genes. Each batch stored a fraction of genome containing different gene sequences, 300bp up- and down-stream sequences, and any intervening sequences (i.e. intergenic regions). Thereafter, each region within each batch was partitioned based on all possible splice donor and acceptor di-nucleotide combinations (i.e., $4^2 \times 4^2 = 256$ combinations). We thus generated a database containing all possible introns. Three introns are illustrated here in black with their corresponding splice donor and acceptor sites highlighted. Around each intron, splice junctions were constructed, using 57bp up- and 57bp down-stream of their flanking sequences (i.e., colored flanking regions). These junctions were stored on the fly, and RNA-seq reads were aligned to these junctions as illustrated, with only unique alignments with no mismatches being retained.

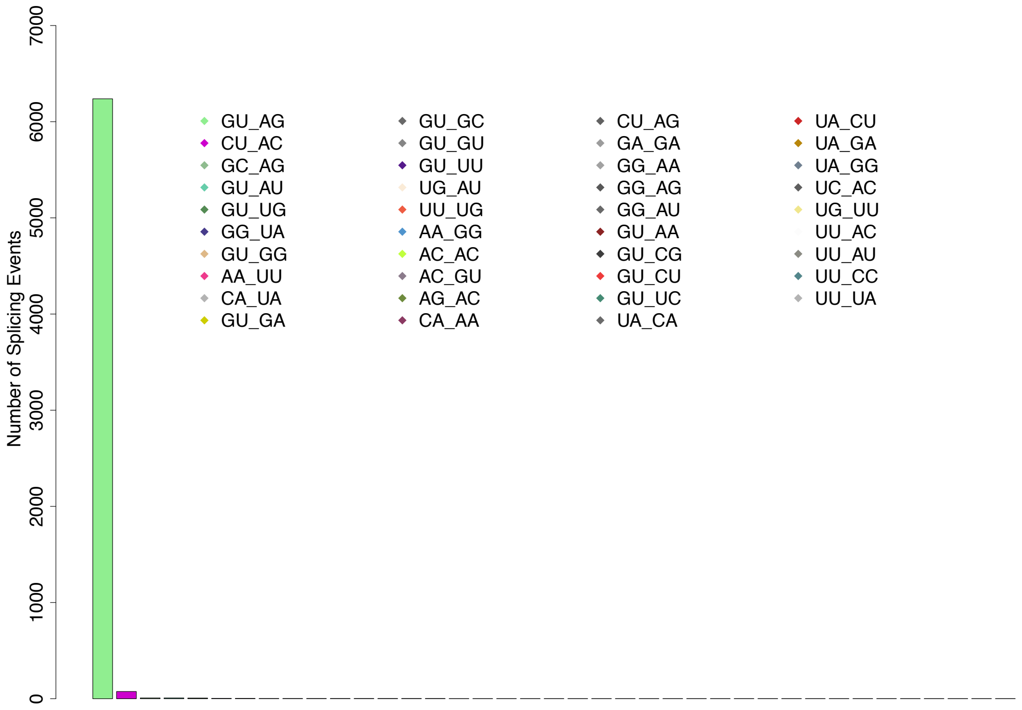

Supplemental Figure S10. Enrichment for 5'-GU and 3'-AG splicing reactions across 116 transcriptomes. To mitigate false mappings, we calculated the False Discovery Rate (FDR) based on results obtained from alignments of 33 samples that were searched against all possible di-nucleotide combinations. FDR was <0.05 when junctions were supported by >13 unique sequence reads starting at different locations along the junctions. This threshold was then applied to junctions obtained from all 116 transcriptomes. Considering only 5'-GU and 3'-AG as splicing signals, applying the threshold we identified 6,238 putative normal splice junctions (5'-GU - 3'-AG) and 105 exon-skipping junctions (Supplemental Table S12).

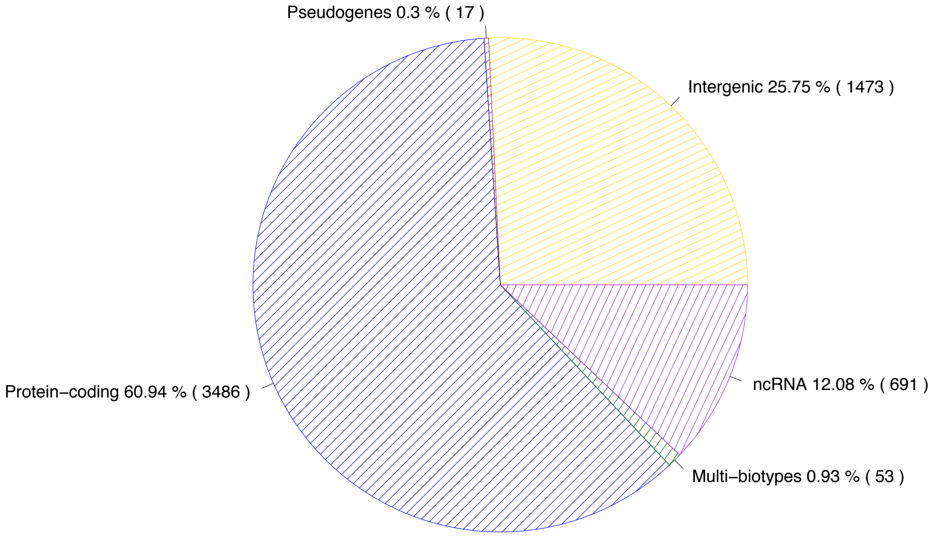

Supplemental Figure S11. Distribution of unannotated introns throughout fission yeast genome. A total of 5,720 unannotated introns are shown here. Multi-biotypes: intron located within overlapping genes/annotations.

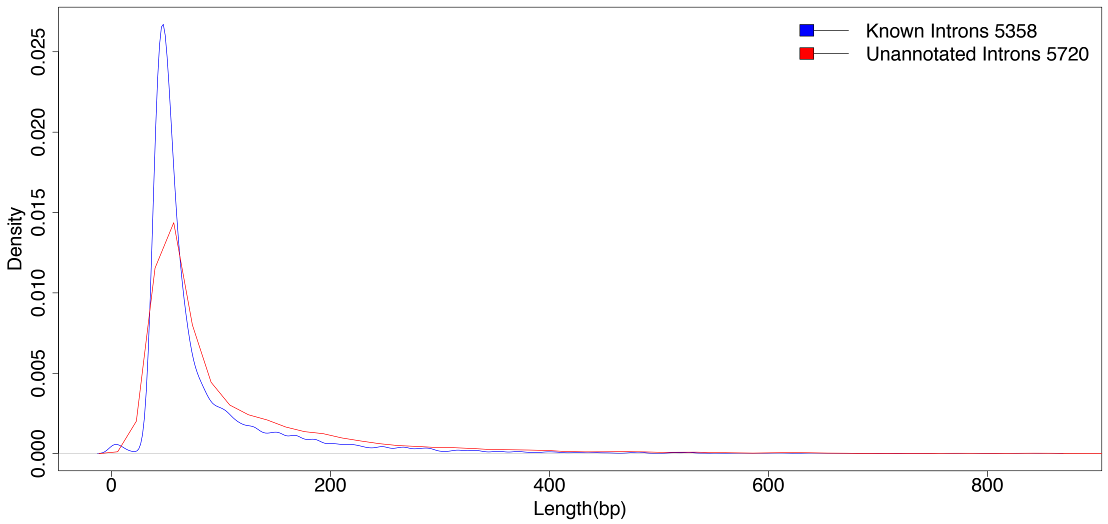

Supplemental Figure S12. Unannotated introns in fission yeast (red) show a comparable length distribution to the one of known introns (blue).

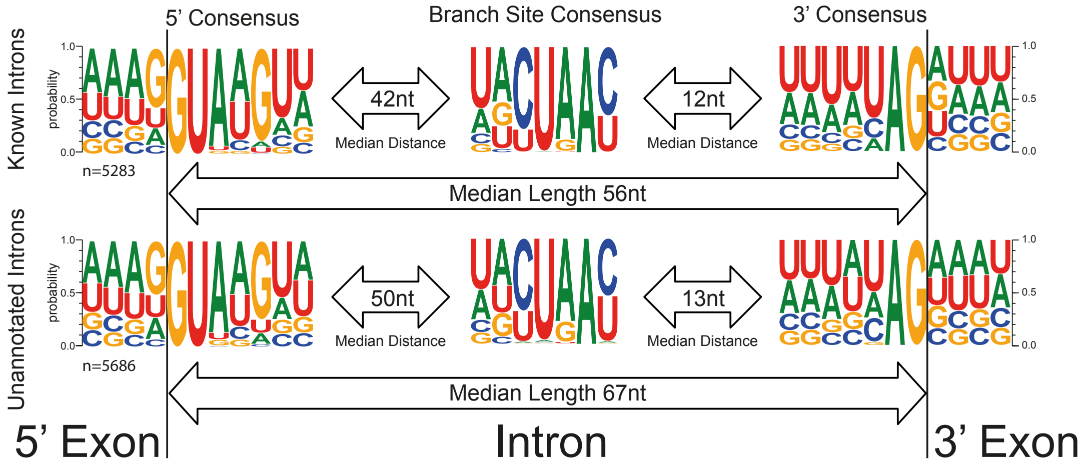

Supplemental Figure S13. Unannotated introns in fission yeast (bottom panel) show similar splicing signal consensus sequences to those of known introns (top panel). Branch-sites were predicted by the FELINES algorithm using default settings (see methods); nt-nucleotids; n-total number of introns used.

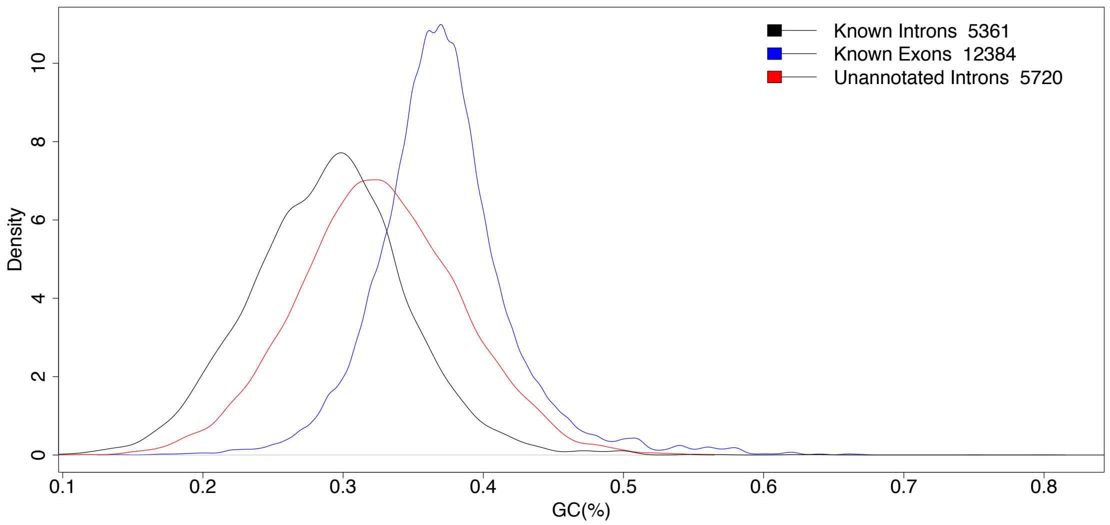

Supplemental Figure S14. Unannotated introns in fission yeast (red) show a different GC content profile to the one of known introns (black), or known annotated exons (blue).

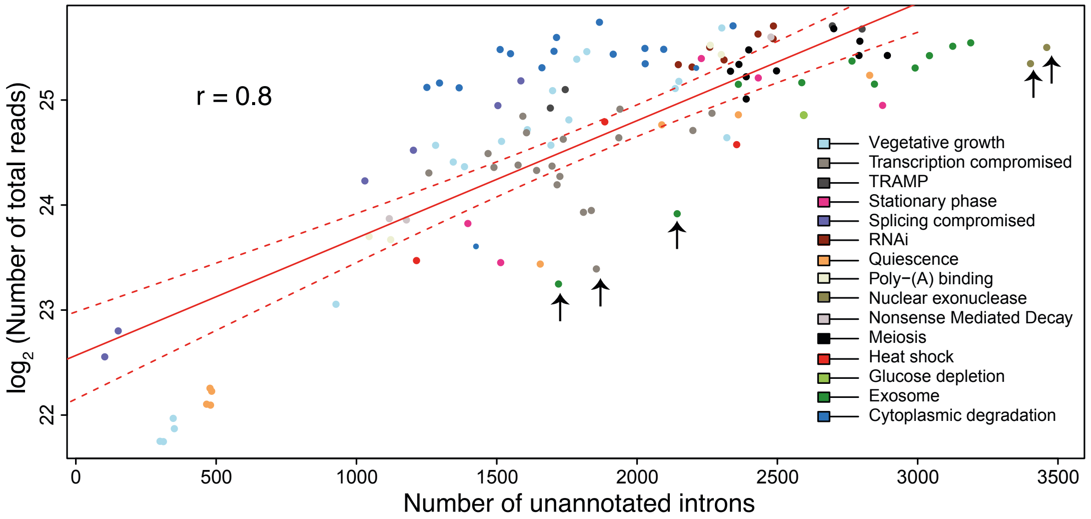

Supplemental Figure S15. Correlation between the samples' sequencing depth and number of novel 5'(GU)-3'(AG) splicing events; r: Pearson's correlation coefficient; red line: fitted regression; dotted red lines: 0.95 confidence levels. Each dot represents a sample (116 in total), grouped according to cellular functions and conditions. Note that a given splicing event could be identified in multiple samples. Arrows highlight accumulation of cryptic events when degradation or transcription regulation is compromised (e.g. *nmt1-dis3* and *dhp1-ts* or *spt6-ts)*.

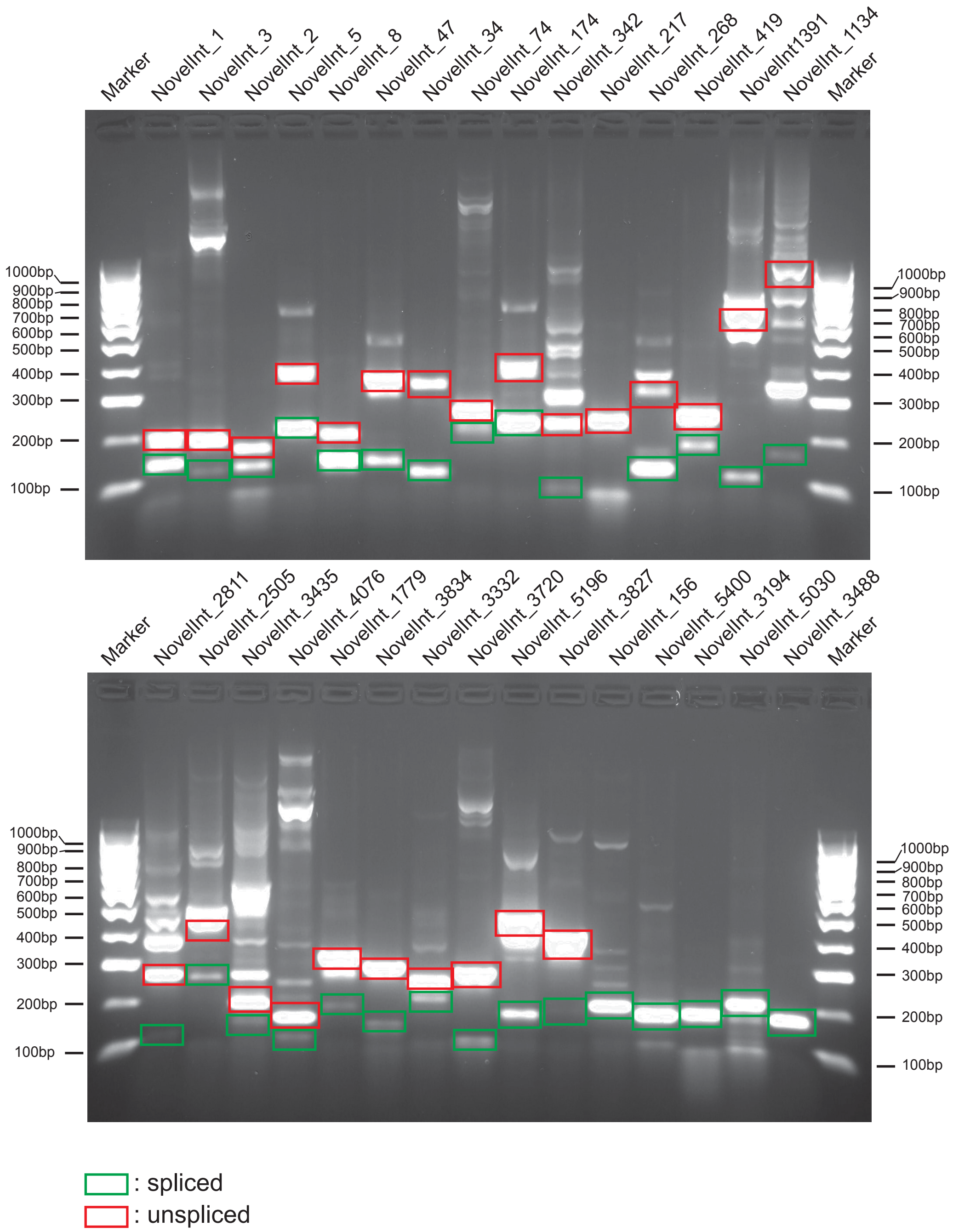

Supplemental Figure S16. Validation of 30 candidate introns by RT-PCR.
A total of 30 unannotated introns of different length and expression levels were randomly selected from the 5,720 introns identified here (23 with two unannotated splice sites and 7 with 1 annotated splice site). Expected unspliced and spliced products are as indicated. Spliced products were further validated by Sanger sequencing (Supplemental Table S13).

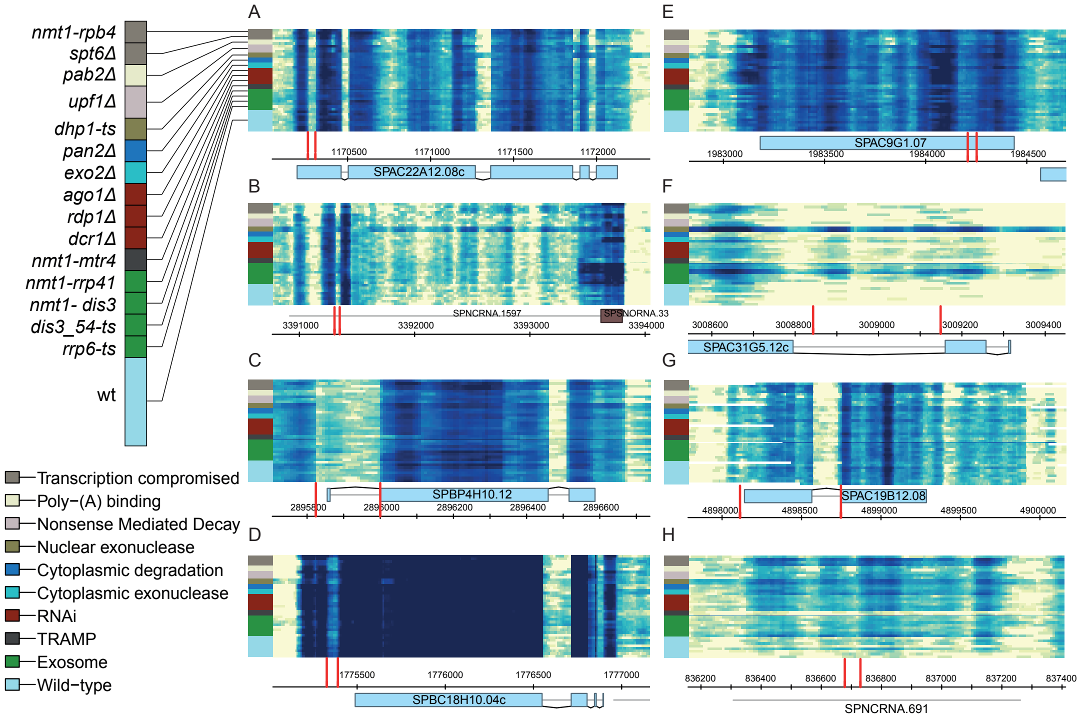

Supplemental Figure S17. Expression profiles of unannotated introns. (**A-D**) Boundaries of unannotated introns (perpendicular red lines) in (**A**) protein-coding gene, (**B**) non-coding RNAs, (**C**) protein-coding gene with likely incorrect annotation, and (**D**) UTR. (**E-H**) Expression profiles of novel-cryptic introns with less obvious boundaries across entire dataset, found within (**E-G**) protein-coding genes or (**H**) non-coding RNAs. Panels **C** and **G** showing examples of 5' cryptic splice-sites linked to an annotated site. Genes that are located above the chromosomal line are found on forward strand and those below the line are on reverse strand. Each row represents a different transcriptome as indicated. A total of 116 transcriptomes were analysed and grouped according to their cellular state or condition tested. Only few samples are shown here for simplicity. A total of 5,720 introns were discovered. Shown are selected introns that were also confirmed by RT-PCR and Sanger sequencing (for a full list, see relevant column in Supplemental Table S13).

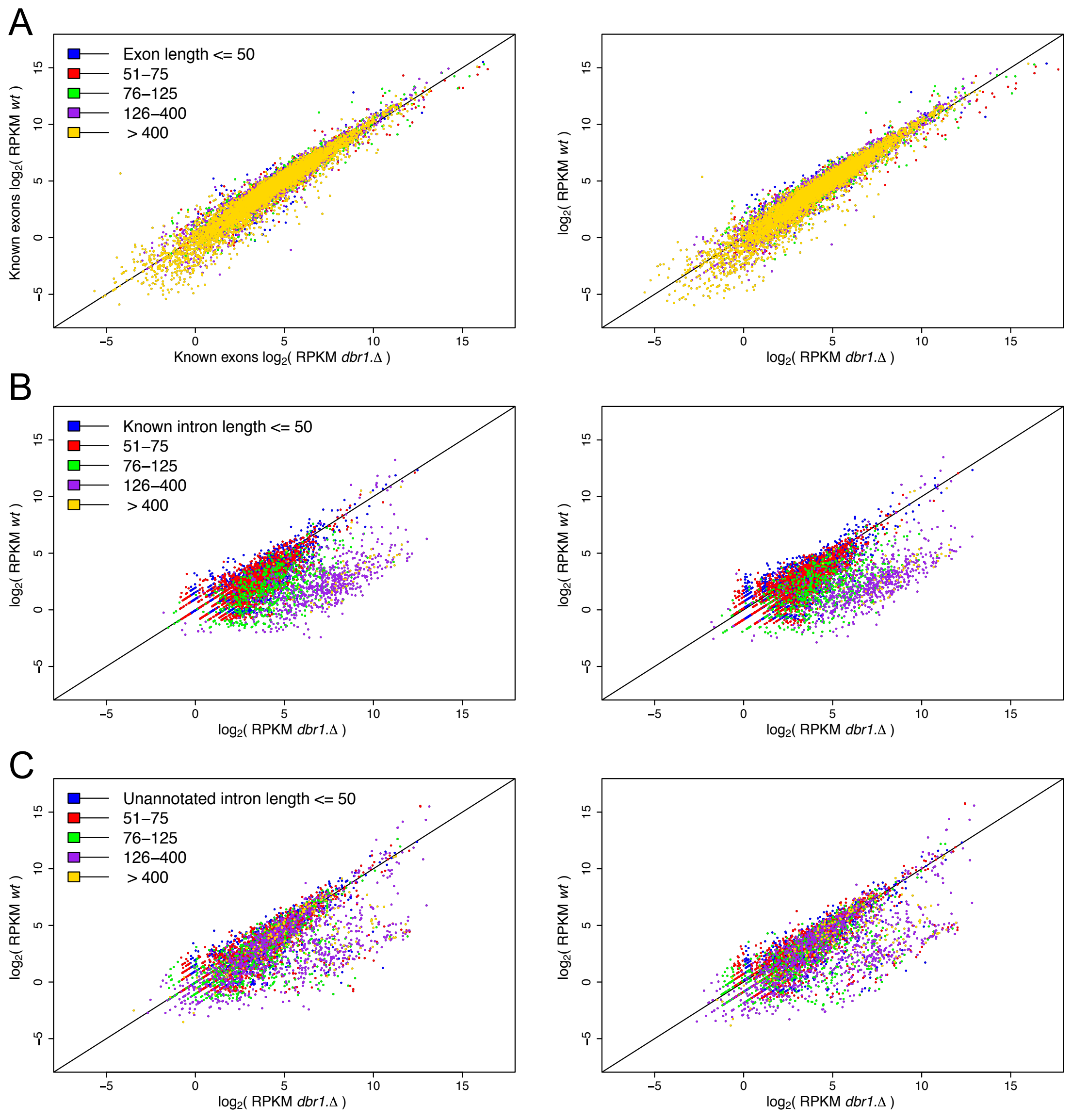

Supplemental Figure S18. Expression profiles of annotated exons, introns and unannotated introns in debranching mutant compared to wild-type.

(**A**) Expression profiles of all annotated exons (12,384) in *dbr1*Δ cells (X axis) and wild-type cells (Y axis), in two biological replicates (left and right panels). Exons are colored by their length as indicated.

(**B**) As in (A) but for all annotated introns (5,361).

(**C**) As in (A) but for all putative unannotated introns identified in this study (5,720).
